# Neural Resources Supporting Language Production vs. Comprehension in Chronic Post-stroke Aphasia: A Meta-Analysis Using Activation Likelihood Estimates

**DOI:** 10.3389/fnhum.2021.680933

**Published:** 2021-10-25

**Authors:** Arianna N. LaCroix, Eltonnelle James, Corianne Rogalsky

**Affiliations:** ^1^College of Health Sciences, Midwestern University, Glendale, AZ, United States; ^2^College of Health Solutions, Arizona State University, Tempe, AZ, United States

**Keywords:** speech, language, production, comprehension, aphasia, meta-analysis, fMRI, stroke

## Abstract

In post-stroke aphasia, language tasks recruit a combination of residual regions within the canonical language network, as well as regions outside of it in the left and right hemispheres. However, there is a lack of consensus as to how the neural resources engaged by language production and comprehension following a left hemisphere stroke differ from one another and from controls. The present meta-analysis used activation likelihood estimates to aggregate across 44 published fMRI and PET studies to characterize the functional reorganization patterns for expressive and receptive language processes in persons with chronic post-stroke aphasia (PWA). Our results in part replicate previous meta-analyses: we find that PWA activate residual regions within the left lateralized language network, regardless of task. Our results extend this work to show differential recruitment of the left and right hemispheres during language production and comprehension in PWA. First, we find that PWA engage left perilesional regions during language comprehension, and that the extent of this activation is likely driven by stimulus type and domain-general cognitive resources needed for task completion. In contrast to comprehension, language production was associated with activation of the right frontal and temporal cortices. Further analyses linked right hemisphere regions involved in motor speech planning for language production with successful naming in PWA, while unsuccessful naming was associated with the engagement of the right inferior frontal gyrus, a region often implicated in domain-general cognitive processes. While the within-group findings indicate that the engagement of the right hemisphere during language tasks in post-stroke aphasia differs for expressive vs. receptive tasks, the overall lack of major between-group differences between PWA and controls implies that PWA rely on similar cognitive-linguistic resources for language as controls. However, more studies are needed that report coordinates for PWA and controls completing the same tasks in order for future meta-analyses to characterize how aphasia affects the neural resources engaged during language, particularly for specific tasks and as a function of behavioral performance.

## Introduction

Aphasia is an acquired communication disorder in which individuals have difficulty with the production and/or comprehension of language, typically following a left hemisphere stroke. Recovery from aphasia is highly variable but largely dependent on stroke specific characteristics such as lesion size and location (particularly the extent of white matter involvement; Turkeltaub, 2019). Each of these characteristics also impacts the neural resources which support language functions post-stroke. For example, large left hemisphere lesions are generally associated with increased right hemisphere activation compared to smaller, more focal left hemisphere lesions (Karbe et al., 1998; Cao et al., 1999; Blasi et al., 2002; Heiss and Thiel, 2006; Sebastian and Kiran, 2011; Griffis et al., 2017; Skipper-Kallal et al., 2017). However, focal damage can also lead to widespread disruptions of functionally and/or anatomically connected brain regions that support language; for example by disrupting critical white matter tracts (Price et al., 2001; Martin et al., 2009; Barwood et al., 2011; Papoutsi et al., 2011; Basilakos et al., 2014; Forkel et al., 2014; Xing et al., 2017, 2018). Both lesion size and location have been associated with behavioral outcomes including overall aphasia severity and specific language abilities (e.g., auditory comprehension, verbal expression; Plowman et al., 2012; Forkel et al., 2014; Marebwa et al., 2017; Xing et al., 2017, 2018; Thye and Mirman, 2018; Benghanem et al., 2019; Turkeltaub, 2019; Wilson and Schneck, 2021). Additionally, the exact neural resources engaged during language tasks in persons with aphasia (PWA) is known to be influenced by task demands, and dependent upon the location of the lesion that resulted in language impairments (Blank et al., 2003; Cherney and Small, 2006; Sebastian and Kiran, 2011; Skipper-Kallal et al., 2017).

Language recovery in post-stroke aphasia is likely driven by a combination of functional reorganization and activation of residual regions within the canonical language network^1^. Functional reorganization involves recruitment of brain regions outside of the canonical language network to varying degrees; these can include left perilesional regions^2^, bilateral cognitive networks, and/or right hemisphere homologs of the left lateralized portions of the canonical language network. While left perilesional regions have consistently been shown to support residual language functions (e.g., Price and Crinion, 2005; Crinion et al., 2006; Warren et al., 2009; Fridriksson et al., 2010; Tyler et al., 2011; Allendorfer et al., 2012; Robson et al., 2014; Griffis et al., 2017; Nenert et al., 2018), the role of the right hemisphere in language recovery is less clear, particularly since right hemisphere activation has been associated with both better (Karbe et al., 1998; Cao et al., 1999; Musso et al., 1999; Blasi et al., 2002; Heiss and Thiel, 2006; Harnish et al., 2008; Raboyeau et al., 2008; Sebastian and Kiran, 2011; Heath et al., 2012; Robson et al., 2014; Griffis et al., 2017; Skipper-Kallal et al., 2017) and poorer language outcomes (Richter et al., 2008; Postman-Caucheteux et al., 2010; Naeser et al., 2011). Small sample sizes and other inconsistencies across studies (e.g., variable tasks and number of trials, differences in thresholding and analysis approaches) likely have contributed to these seemingly mixed results, leaving many unanswered questions regarding neural reorganization and language recovery in aphasia.

Activation likelihood estimation (ALE) is a meta-analysis technique that can be used to overcome the limitations of individual experiments. ALE can identify brain regions which are consistently activated across all imaging studies of interest. The algorithm then determines whether this convergence is higher than what would be expected from a spatially random distribution (Eickhoff et al., 2009). In their 2011 ALE meta-analysis of activation in PWA during any type of language task, Turkeltaub and colleagues found PWA activate a combination of spared regions within the canonical language network, left perilesional regions, and right hemisphere homologs during language (Turkeltaub et al., 2011). A secondary analysis comparing PWA with and without lesions to the left inferior frontal gyrus (IFG) revealed those with lesions to the left IFG activate the right IFG more during language tasks than those with a lesion sparing the left IFG. These results provide some evidence that anatomically and/or functionally homologous regions may be engaged to support language through compensatory processes in PWA. For example, the right inferior frontal gyrus may be more engaged during language tasks when the left inferior frontal gyrus is lesioned, due to shared domain-general cognitive functions (Ries et al., 2016).

However, Turkeltaub et al.'s (2011) meta-analysis almost exclusively included production tasks (75%, 9/12 studies; e.g., picture naming, verb generation) and did not consider possible differences in functional reorganization for production vs. comprehension. Thus, this approach likely missed important insights into the relative contributions of perilesional vs. right hemisphere engagement for language processing post-stroke; particularly since expressive and receptive language processes are typically supported by distinct (but interacting) neural resources that have different lateralization patterns. For instance, language production is associated with a left lateralized dorsal stream in frontal and parietal cortices, while language comprehension is generally supported by bilateral ventral streams in temporal and parietal regions (Hickok and Poeppel, 2007; Friederici, 2011; Bornkessel-Schlesewsky and Schlesewsky, 2013). It is therefore likely that the left and right hemispheres are recruited differently for expressive and receptive language following a left hemisphere stroke. Thus, the seemingly mixed results across individual studies may actually represent separate functional reorganization patterns for productive vs. receptive language following a left hemisphere stroke. For example, damage to the left dorsal stream may result in activation of residual left hemisphere tissue during language production tasks (Fridriksson et al., 2010; Allendorfer et al., 2012), while other studies show recruitment of their right hemisphere homologs (Karbe et al., 1998; Cao et al., 1999; Harnish et al., 2008; Raboyeau et al., 2008; Heath et al., 2012; Skipper-Kallal et al., 2017). For the more bilateral ventral stream, some previous work in PWA suggests increased right ventral stream activation during language comprehension tasks (Crinion and Price, 2005; Crinion et al., 2006), whereas other studies have found activation predominately within the perilesional tissue of the left ventral stream, or even activation of domain-general regions in right frontal cortex (Warren et al., 2009; Tyler et al., 2011; Robson et al., 2014; Griffis et al., 2017; Nenert et al., 2018). Thus, there is a need to conduct meta-analyses investigating language production and comprehension studies separately in order to better understand how functional reorganization may differ for expressive vs. receptive language abilities, particularly regarding perilesional vs. right hemisphere involvement.

Several more recent reviews have also taken critical steps toward summarizing the literature related to the neural resources supporting language functions following a left hemisphere stroke (Hartwigsen and Saur, 2019; Stefaniak et al., 2019; Turkeltaub, 2019; Wilson and Schneck, 2021). Two of these papers primarily focus on providing comprehensive reviews of theories related to language recovery from post-stroke aphasia (Stefaniak et al., 2019; Turkeltaub, 2019), and also how different person- and stroke-specific characteristics impact language recovery (Turkeltaub, 2019). Hartwigsen and Saur's (2019) review primarily focuses on longitudinal studies of language recovery and how neurostimulation may impact language recovery in the acute and sub-acute recovery stages. In a partial extension of this work, Wilson and Schneck (2021) conducted a systematic review in which they critically appraised the strength of the literature regarding the neural resources supporting language in the chronic stage of aphasia recovery. Wilson and Schneck (2021) also sought to identify brain regions that were associated with increased or decreased activation for PWA during language tasks. While their review and analysis is incredibly informative, it included multiple methods for reporting findings (i.e., coordinates, figures, text descriptions were all included), thus, the spatial resolution of their findings of activation (or deactivation) is somewhat limited. Furthermore, activation and deactivations were not discussed in terms of differences between expressive and receptive language processes (Wilson and Schneck, 2021). Therefore, questions remain regarding the neural resources supporting language production vs. comprehension in post-stroke aphasia.

Comparing the neural resources which are engaged by language production and comprehension may be helpful in characterizing the mechanisms which drive right hemisphere engagement during language in post-stroke aphasia. A direct comparison of language production and comprehension tasks in post-stroke aphasia is warranted since several individual studies indicate that production and comprehension recruit partially distinct neural resources in PWA (Léger et al., 2002; Cherney and Small, 2006; Eaton et al., 2008; Richter et al., 2008; Sebastian and Kiran, 2011; Haldin et al., 2018). This direct comparison can also help elucidate whether the right hemisphere is engaged in language in PWA due to speech and language-specific processes or more domain-general cognitive processes, which are known to recruit similar, yet distinct neural resources as language (e.g., Fedorenko et al., 2012). For example, voxels in the right hemisphere which are activated by production and also more significantly activated for production than comprehension, may be performing similar computations as regions within the left dorsal stream, including those involved in motor speech planning for language. Alternatively, finding right hemisphere regions that are equally activated by both production and comprehension, may instead suggest that the right hemisphere is engaged by language through domain-general processes related to attention, executive control, or alertness, or perhaps through language resources shared by production and comprehension tasks (e.g., phonological processes, decision-making).

The present meta-analysis expands upon previous reviews by using activation likelihood estimation (ALE; Turkeltaub et al., 2002; Eickhoff et al., 2009), which provides greater spatial resolution than region of interest approaches, to separately investigate the neural resources engaged by language production vs. comprehension in persons with chronic aphasia. This direct comparison between production and comprehension is now sufficiently powered (Eickhoff et al., 2016) due to the substantial increase in published fMRI/PET studies over the last 10 years. We focus on chronic aphasia here as longitudinal studies of language recovery in aphasia indicate that in the acute stage (<6 months post-stroke), there is a gradual transition from the initial recruitment of right hemisphere resources to left perilesional regions as language recovers (Saur et al., 2006; Nenert et al., 2018; Hartwigsen and Saur, 2019). The present meta-analysis sought to determine what neural resources are consistently engaged across studies by language production and/or comprehension in PWA, and how this compares to controls. We additionally aimed to explore whether the neural resources engaged by language differ for specific tasks (e.g., picture naming, word generation) in PWA and controls.

## Methods

### Literature Search

**Figure 1:**
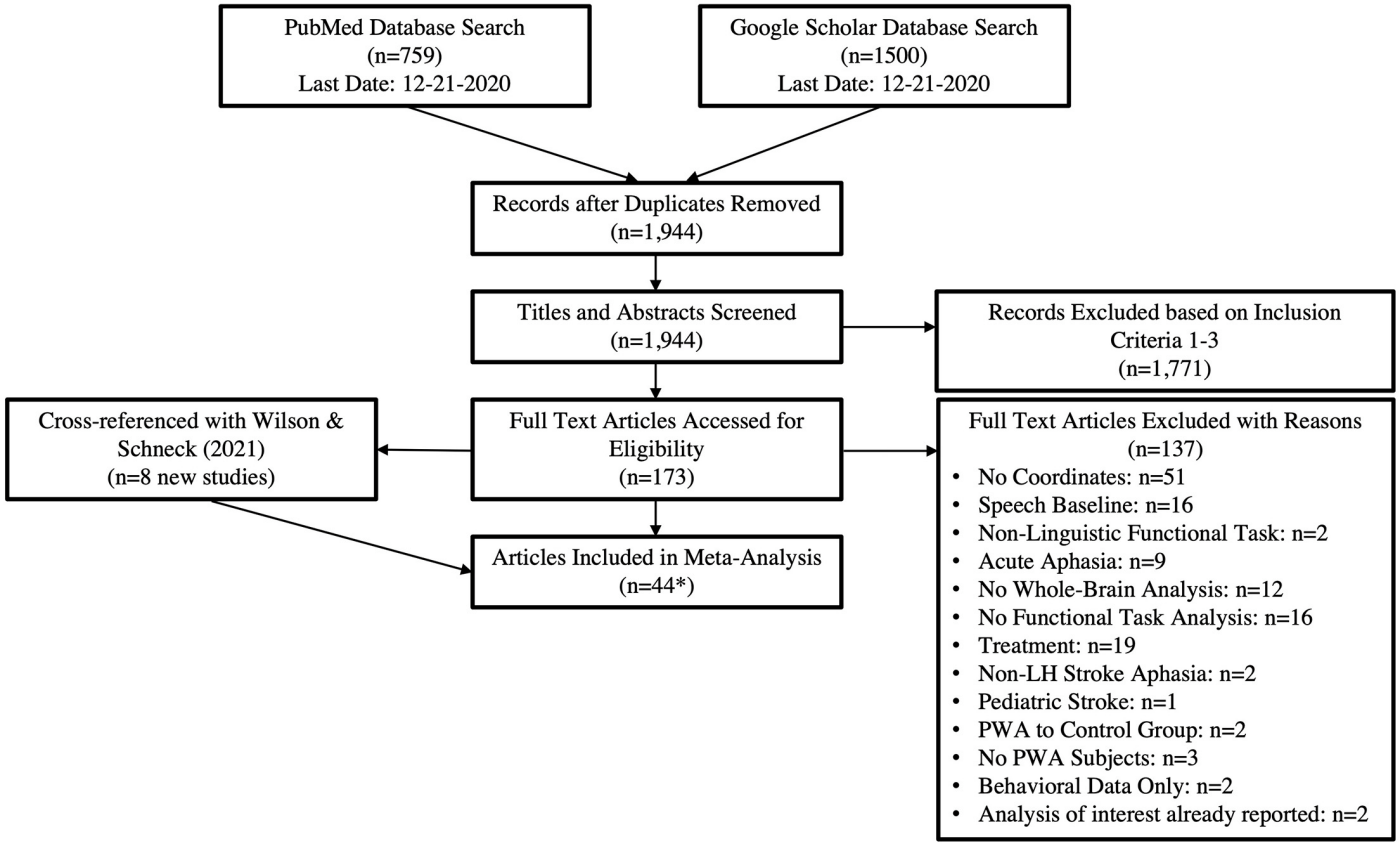
The PRISMA flow diagram adapted for our literature search. ^*^The 44 articles resulted in 49 tasks as five papers included both production and comprehension tasks. This resulted in 29 production and 21 comprehension tasks being included in the analyses.

This meta-analysis was conducted following the Preferred Reporting Items for Systematic Reviews and Meta-Analyses (PRISMA) framework (Moher et al., 2009). A PRISMA flow diagram for the literature search is documented in Figure 1. The search criteria for each database are reported in Table 1. PubMed and Google Scholar were periodically searched between August 2015 and December 2020 to locate peer-reviewed articles published prior to December 2020 that used fMRI or PET to measure brain activations to speech and language stimuli in PWA. The PubMed search yielded a total of 759 articles. For Google Scholar, we extracted the first 150 citations for each search criteria, which resulted in 1,500 citations (150 citations x 10 search criteria). The combined lists resulted in 1,944 articles after duplicates were removed. The titles and abstracts of these 1,944 articles were reviewed to determine if they met the following criteria: (1) publication was written in English, (2) adult participants with a history of stroke, and (3) use of fMRI or PET methodologies. We identified 173 citations meeting these three criteria and extracted the full-text articles for further review. Of the 173 citations meeting our first three criteria, 137 were excluded for the reasons reported in Figure 1 and Supplementary Table 1. This left 36 articles which met our additional inclusion criteria: (4) studies reported peak coordinates from a whole-brain analysis, (5) compared language production or comprehension tasks to a non-speech baseline (e.g., rest, noise, tones, visual stimuli), (6) in persons with chronic aphasia (>6 months post-stroke). In addition, we manually searched Wilson and Schneck's (2021) recent systematic review and meta-analysis of functional neuroimaging studies in post-stroke aphasia and identified an additional eight articles meeting our inclusion criteria^3^.

**Table 1:** Search terms for each database.

**Database**	**Search terms**
PubMed Google Scholar	speech production aphasia fMRI; speech comprehension aphasia fMRI; speech processing aphasia fMRI; expressive language aphasia fMRI; receptive language aphasia fMRI; speech production aphasia PET; speech comprehension aphasia PET; speech processing aphasia PET; expressive language aphasia PET; receptive language aphasia PET

Our combined searches resulted in a total of 44 articles being included in the meta-analysis (Supplementary Table 6). From these 44 articles, we extracted 50 tasks, 29 production (207 PWA and 194 control subjects) and 21 comprehension tasks (228 PWA and 207 control subjects) for analysis; five articles included both production and comprehension tasks. Of these 50 tasks, 33 tasks, 16 production (164 PWA and 194 control subjects) and 17 comprehension (205 PWA and 207 control subjects), included PWA and control participants completing the same task in the same study; these tasks were analyzed separately to compare activations between the two groups. Since not all studies included control data, each section within the results is structured to first present ALEs that included all PWA data, followed by ALEs for the control participants, and lastly group comparisons using the subset of data that included both PWA and controls in the same study completing the same task. Production tasks were overt or covert and included picture naming, word generation (noun, verb), repetition (syllable, word), and spontaneous language production. Comprehension tasks could be auditory or visual and included listening to sentences, semantic decision, reading (single words, pseudowords), and rhyming tasks.

### Activation Likelihood Estimate Meta-Analysis

Activation likelihood estimates (ALEs), a coordinate-based meta-analysis method, were calculated for each single condition and contrast of interest using GingerALE Version 3.0.2 (Turkeltaub et al., 2002; Eickhoff et al., 2009, 2012). All MNI coordinates were transformed into Talairach space using GingerALE's stereotaxic coordinate converter. Talairach coordinates were then combined to create 3D maps depicting the likelihood of activation within each voxel in a MRI template. Significant areas were identified depending on whether the identified area was more likely to occur in comparison to spatially random distributions. Number of subjects in each study was input into the analysis and used to calculate the amount of blurring and uncertainty around the coordinates, which is accounted for in the FWHM of the Gaussian curve. It is also recommended that ALEs include at least 8–17 studies to avoid clusters that are primarily driven by one study (Eickhoff et al., 2016). Single condition analyses were thresholded using a cluster-level correction for multiple comparisons at *p* = 0.05 (10,000 permutations) with a cluster forming threshold of *p* < 0.001 (Eickhoff et al., 2017). The following single condition analyses were computed separately for PWA and controls: a combined analysis for language production and comprehension, language production only, and language comprehension only. We additionally computed separate sub-analyses for PWA and controls using coordinates associated with tasks frequently used to measure language abilities in PWA: picture naming, auditory sentence listening, semantic decisions, and word generation (Wilson and Schneck, 2021).

Contrast analyses were computed to identify common and distinct neural resources involved in language production and comprehension within PWA and controls (within-subject analyses). Contrast analyses were also used to conduct between-group comparisons for the combined language production and comprehension, language production only, language comprehension only, and four task-specific analyses (picture naming, auditory sentence listening, semantic decisions, and word generation). Contrast analyses use ALE maps thresholded for multiple comparisons, therefore the contrast threshold was set to uncorrected *p* = 0.05 (10,000 permutations) with a 200 mm^3^ minimum volume (Eickhoff et al., 2016). ALE statistical maps were rendered onto the ch2.nii template brain using MRIcron (Rorden and Brett, 2000).

## Results

### Neural Resources Engaged by Language

We first computed an ALE which combined language production and comprehension in a single analysis to identify neural resources which are generally engaged by language in PWA and controls, regardless of task.

#### All PWA

**Figure 2:**
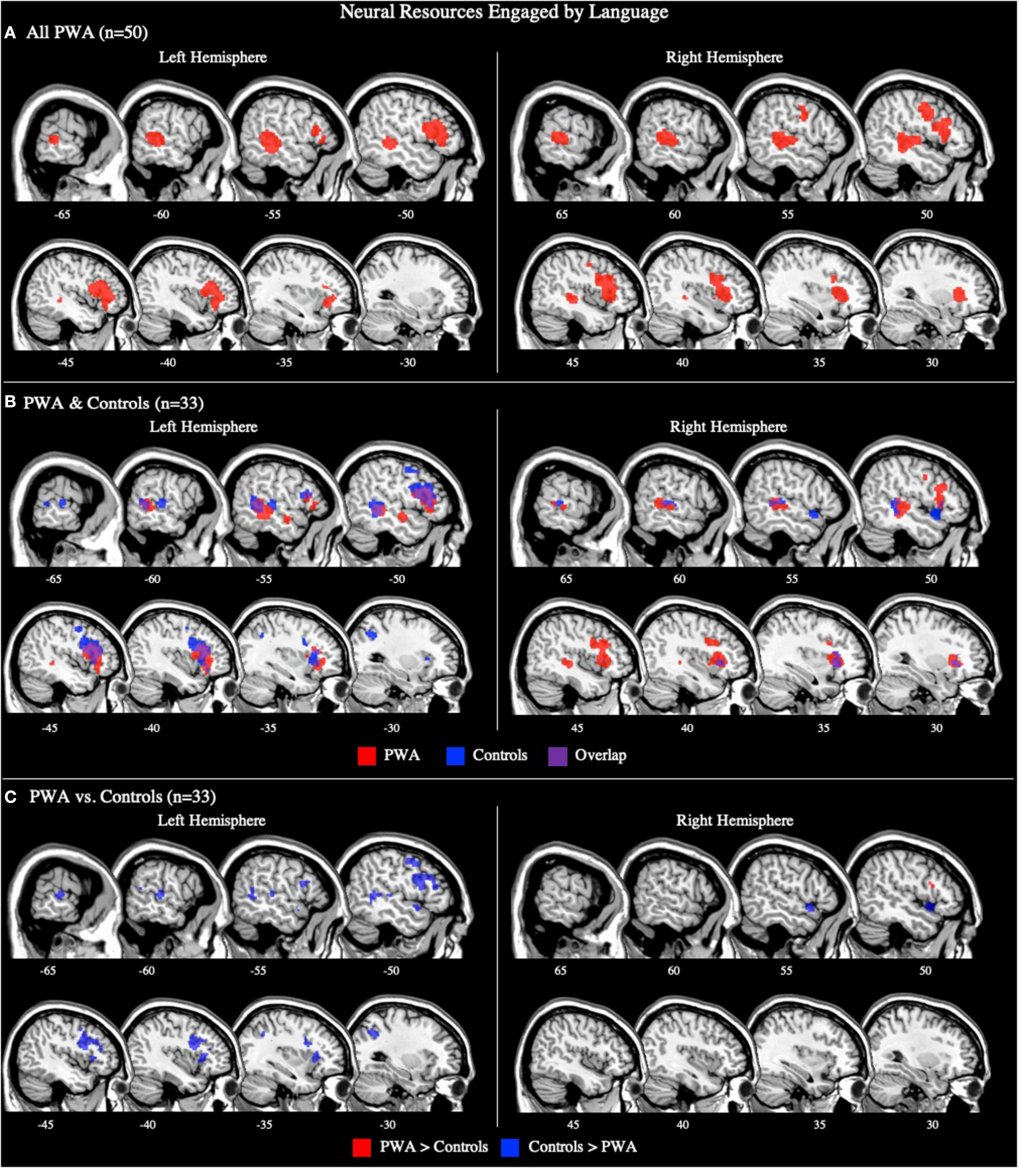
Representative sagittal slices for the combined production and comprehension ALEs in **(A)** PWA (*p* < 0.001 corrected), **(B)** PWA and controls (*p* < 0.001 corrected), and **(C)** PWA vs. controls (*p* = 0.05 uncorrected). **(B,C)** only include data from tasks which included both PWA and control data. Sample size denotes the number of tasks included in the ALE.

The combined ALE included all 50 tasks (29 production, 21 comprehension) and identified five significant clusters for PWA in bilateral fronto-temporal regions. Peak activations within the temporal lobe included the right posterior superior temporal gyrus and left posterior middle temporal gyrus. In the frontal lobe, peak activations were in the left superior and middle frontal gyri, as well as the right anterior insula (largest cluster). These peak activations additionally extended into the bilateral inferior frontal gyri, left medial frontal gyrus, left cingulate gyrus, left posterior insula, and right middle and precentral gyri (*p* < 0.001 corrected; Table 2; Figure 2A). We also conducted this analysis using the 33 tasks that included PWA and control data; the coordinates associated with this analysis for PWA are reported in Supplementary Table 2 and depicted in Figure 2B.

**Table 2:** Anatomical locations of peak coordinates and cluster size for each single condition and contrast ALE in PWA.

**Condition**	**Anatomical location**	**Peak coordinates**	**Voxels (mm^**3**^)**
**Single condition ALEs**			
Production and	Left middle frontal gyrus^*^, left inferior frontal gyrus (pars orbitalis), left insula	−44, 22, 20	13,704
Comprehension	Left superior frontal gyrus^*^, left cingulate gyrus, right medial frontal gyrus, left medial frontal gyrus	−6, 8, 50	6,760
	Left middle temporal gyrus^*^	−50, −36, −2	5,736
	Right insula^*^, right precentral gyrus, right middle frontal gyrus, right inferior frontal gyrus (pars triangularis)	32, 20, 6	16,376
	Right superior temporal gyrus^*^	58, −34, 6	8,936
Production	Left middle frontal gyrus^*^	−42, 26, 18	1,248
	Left cingulate gyrus^*^, right medial frontal gyrus, right superior frontal gyrus	−8, 10, 40	3,880
	Left middle temporal gyrus^*^	−60, −34, 4	1,920
	Right inferior frontal gyrus (pars triangularis^*^), right precentral gyrus, right insula	46, 18, 10	11,144
	Right superior temporal gyrus^*^	58, −34, 6	8,912
Comprehension	Left inferior frontal gyrus (pars orbitalis)^*^	−38 28 −4	2,400
	Left middle frontal gyrus^*^, left insula	−46, 20, 20	4,168
	Left medial frontal gyrus^*^	−8, 8, 50	1,408
	Left precentral gyrus^*^	−46, −2, 40	1,488
	Left middle temporal gyrus^*^, left superior temporal gyrus	−50, −36, −2	3,368
	Left middle temporal gyrus^*^	−40, −64, 20	968
	Left superior temporal gyrus^*^	−50, −8, −10	1,848
	Right middle frontal gyrus^*^	46, 22, 26	1,088
	Right claustrum^*^, right inferior frontal gyrus (pars orbitalis)	30, 20, 4	2,640
**Production vs. comprehension contrast ALEs**			
Conjunction analysis	Left middle frontal gyrus^*^	−42, 24, 18	776
	Left superior frontal gyrus^*^	−2, 6, 52	56
	Left medial frontal gyrus^*^	−6, 10, 46	80
	Left middle temporal gyrus^*^	−60, −40, 6	256
	Left middle temporal gyrus^*^	−56, −32, −2	8
	Left middle temporal gyrus^*^	−52, −32, 2	8
	Right insula^*^, right inferior frontal gyrus (pars orbitalis)	32, 20, 6	1,416
Production > Comprehension	Right precentral gyrus^*^	51.3, −12.3, 33	2,440
	Right superior temporal gyrus^*^	56.8, −30.9, 10.4	6,408
Comprehension > Production	Left inferior frontal gyrus (pars triangularis,^*^ pars orbitalis)	−46.8, 20.7, 14	9,000
	Left superior frontal gyrus^*^	−10, 10, 54	1,248
	Left precentral gyrus^*^, left middle frontal gyrus	−43.3, −1.1, 46.2	1,536
	Left middle temporal gyrus^*^	−53, −38, −8	3,288
	Left middle temporal gyrus^*^	−36, −65, 24	968
	Left superior temporal gyrus^*^, left middle temporal gyrus	−49.6, −5.8, −11.9	1,848
	Right middle frontal gyrus^*^	42, 18, 24	1,104
	Right putamen (lentiform nucleus)^*^, right inferior frontal gyrus (pars orbitalis), right insula	26, 16, 3	944

*The x, y, z coordinates are in Talairach space and refer to the peak voxel activated in each cluster. All single condition ALEs are thresholded at p < 0.001 corrected and contrast ALEs at p = 0.05 uncorrected. Asterisks indicate anatomical location of peak voxel*.

#### Controls

The combined language production and comprehension ALE for controls included 33 tasks (16 production, 17 comprehension). This ALE identified seven significant clusters in controls. The largest cluster's peak was in the left inferior frontal gyrus (pars opercularis). Subsequent peaks were identified in the left superior frontal gyrus, left precentral gyrus, left middle temporal gyrus, left superior temporal gyrus, right superior temporal gyrus (two peaks), and left superior parietal lobule (*p* < 0.001 corrected; Supplementary Table 3; Figure 2B).

#### PWA vs. Controls

The ALE conjunction analysis using the 33 tasks which included PWA and control data revealed nine significant clusters activated in PWA and controls. Six clusters were in the left hemisphere with the largest peak being in the left inferior frontal gyrus (pars triangularis). Smaller activations were found in the left superior frontal gyrus, left medial frontal gyrus, and left posterior superior temporal gyrus (three peaks). The remaining three peaks were in the right hemisphere and included the right medial frontal gyrus, right anterior insula, and right posterior middle temporal gyrus. As expected, the contrast analysis revealed controls activate the canonical language network more than PWA (likely at least in part due to PWA having lesions in these left perisylvian areas). The largest peak activation for controls compared to PWA was found in the left inferior frontal gyrus (pars opercularis), with smaller activations being identified in the left inferior frontal gyrus (pars triangularis), left superior parietal lobule, left anterior and posterior superior temporal gyrus, left posterior middle temporal gyrus, right medial frontal gyrus, and right anterior superior temporal gyrus. Conversely, PWA, compared to controls, only activated the right inferior frontal gyrus (pars triangularis) more during all language tasks (*p* = 0.05 uncorrected; Table 3; Figure 2C).

**Table 3:** Anatomical locations of peak coordinates and cluster size for each contrast ALE comparing PWA and controls.

**Condition**	**Anatomical location**	**Peak coordinates**	**Voxels (mm^**3**^)**
**Production and comprehension**			
Conjunction analysis	Left inferior frontal gyrus (pars triangularis)^*^, left middle frontal gyrus	−44, 28, 16	7,608
	Left medial frontal gyrus^*^	−6, 14, 44	8
	Left superior frontal gyrus^*^	−4, 6, 54	768
	Left superior temporal gyrus^*^, left middle temporal gyrus	−56, −42, 6	2,544
	Left superior temporal gyrus^*^	−56, −24, 0	24
	Left superior temporal gyrus^*^	−56, −26, 2	24
	Right insula^*^, right inferior frontal gyrus (pars orbitalis, pars triangularis)	36, 22, 0	1,944
	Right medial frontal gyrus^*^	8, 8, 46	8
	Right middle temporal gyrus^*^, right superior temporal gyrus	54, −36, 4	1,232
PWA > Controls	Right inferior frontal gyrus (pars triangularis^*^)	54, 14, 20	208
Controls > PWA	Left inferior frontal gyrus (pars opercularis)^*^, left middle frontal gyrus, left precentral gyrus	−42.6, 10, 26	7,832
	Left inferior frontal gyrus (pars triangularis)^*^	−36, 24, 6	1,408
	Left middle temporal gyrus^*^, left superior temporal gyrus	−50, −46, 4	1,104
	Left superior temporal gyrus^*^	−60, −26, 8	1,040
	Left superior temporal gyrus^*^	−53, 8, −8	384
	Left superior parietal lobule^*^	−32, −60, 42	1,376
	Right medial frontal gyrus^*^, left superior frontal gyrus, left medial frontal gyrus	2.2, 16.9, 46.4	3,064
	Right superior temporal gyrus^*^	54, 12, −6	1,192
**Production**			
Conjunction analysis	Left middle frontal gyrus^*^	−42, 26, 18	1,112
	Right insula^*^, right inferior frontal gyrus (pars triangularis)	36, 22, 2	632
	Right superior temporal gyrus^*^, right middle temporal gyrus	62, −26, 2	1,056
Controls > PWA	Left inferior frontal gyrus (pars opercularis^*^, pars triangularis), left insula	−49.2, 12.8, 21.2	7,720
	Left superior frontal gyrus^*^, left medial frontal gyrus	−2.2, 15.7, 52.3	3,648
	Left middle temporal gyrus^*^, left superior temporal gyrus	−54, −44, 2	2,232
	Right inferior frontal gyrus (pars triangularis^*^)	54, 22, 2	240
	Right middle temporal gyrus^*^	48, −40, 8	408
	Right superior temporal gyrus^*^	62, −26, 8	616
	Right superior temporal gyrus^*^	54, 12, −6	496
**Comprehension**			
Conjunction Analysis	Left inferior frontal gyrus (pars orbitalis)^*^	−40, 30, −10	1,168
	Left inferior frontal gyrus (pars opercularis)^*^, left middle frontal gyrus	−44, 14, 22	768
	Left inferior frontal gyrus (pars triangularis)^*^	−46, 22, 12	584
	Left middle temporal gyrus^*^	−54, −40, 0	2,136
PWA > Controls	Left precentral gyrus^*^	−47, −6, 38	688
Controls > PWA	Left middle frontal gyrus^*^	−42, 12, 30	848

*The x, y, z coordinates are in Talairach space and refer to the peak voxel activated in each cluster. All contrast ALEs are thresholded at p = 0.05 uncorrected. Asterisks indicate anatomical location of peak voxel*.

### Neural Resources Engaged by Language Production

#### All PWA

**Figure 3:**
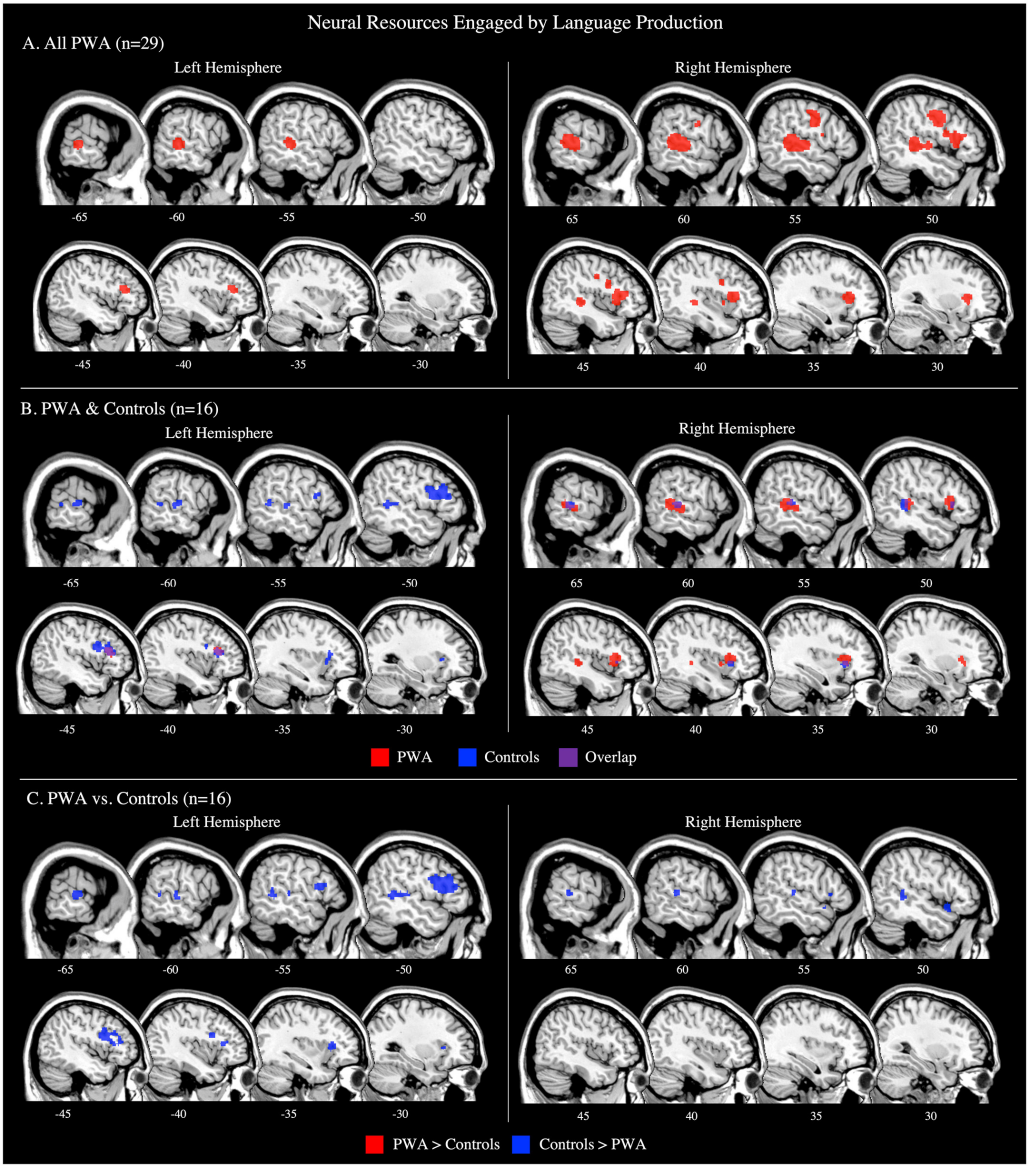
Representative sagittal slices for the language production ALEs in **(A)** PWA (*p* < 0.001 corrected), **(B)** PWA and controls (*p* < 0.001 corrected), and **(C)** PWA vs. controls (*p* = 0.05 uncorrected). **(B,C)** only include data from tasks which included both PWA and control data. Sample size denotes the number of tasks included in the ALE.

The language production ALE included all 29 production tasks with aphasia data. We also conducted this analysis using the 16 production tasks that included PWA and control data; the coordinates associated with this analysis for PWA are reported in Supplementary Table 2 and depicted in Figure 3B. The ALE with all 29 production tasks identified five significant clusters in PWA. The largest peak was in the right inferior frontal gyrus (pars triangularis). Smaller peaks were identified in the right posterior superior temporal gyrus, left cingulate gyrus, left middle frontal gyrus, and left posterior middle temporal gyrus. These peak activations extended into the right precentral gyrus, right superior frontal gyrus, right medial frontal gyrus, and right anterior insula (*p* < 0.001 corrected; Table 2; Figure 3A).

We additionally decided to compute *post-hoc* ALE analyses for each aphasia subtype. However, due to a paucity of aphasia type-specific activations reported in literature, *post-hoc* ALEs were only computed for the two most frequent aphasia types for which data was reported: Broca's aphasia (7 studies with 15 PWA) and Wernicke's aphasia (3 studies with 8 PWA). However, these ALEs should be interpreted with caution due to having fewer than eight studies included. The findings from both the Broca's and Wernicke's aphasia ALEs align with the production results from the larger PWA sample. The Broca's aphasia ALE identified four significant clusters with peaks in the right precentral gyrus (largest peak), right middle frontal gyrus, right medial frontal gyrus, and left postcentral gyrus. The Wernicke's aphasia ALE identified three significant clusters with peaks in the right inferior frontal gyrus (pars opercularis; largest), right middle temporal gyrus, and left caudate (Supplementary Table 4; Supplementary Figure 1).

#### Controls

Sixteen production tasks included control data. Six clusters were significantly activated by controls during language production. The control group's largest peak was in the left inferior frontal gyrus (pars opercularis). Additional peaks were identified in the left superior frontal gyrus, left middle temporal gyrus, left superior temporal gyrus, right insula, and right superior temporal gyrus (*p* < 0.001 corrected; Supplementary Table 3; Figure 3B).

#### PWA vs. Controls

Sixteen production studies included PWA and controls completing the same task. Three significant clusters were activated by both PWA and controls during language production: peak activations were identified in the left middle frontal gyrus (largest cluster), right posterior superior temporal gyrus, and right anterior insula. PWA did not significantly activate any regions more than controls during language production. However, the analysis identified seven clusters significantly more active in controls than PWA: the largest cluster was in the left inferior frontal gyrus (pars opercularis), and smaller clusters were identified in the left superior frontal gyrus, bilateral posterior middle temporal gyri, right anterior superior temporal gyrus, and right inferior frontal gyrus (pars triangularis; *p* = 0.05 uncorrected; Table 3; Figure 3C). It was not possible to compare persons with Broca's aphasia or persons with Wernicke's aphasia to controls completing the same task as there were only two studies for each aphasia diagnosis that also included control data for the same task.

### Neural Resources Engaged by Language Comprehension

#### All PWA

**Figure 4:**
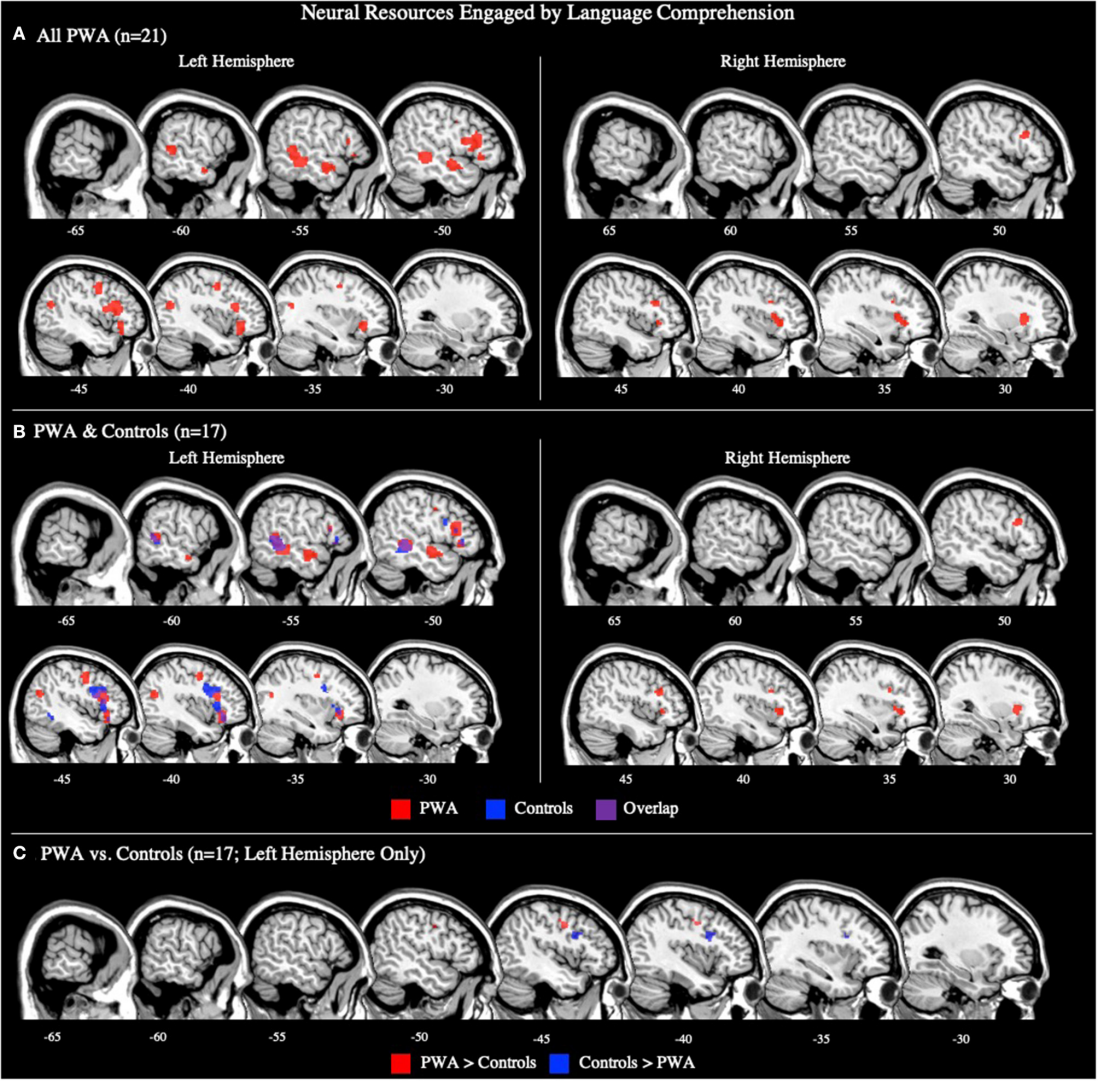
Representative sagittal slices for the language comprehension ALEs in **(A)** PWA (*p* < 0.001 corrected), **(B)** PWA and controls (*p* < 0.001 corrected), and **(C)** PWA vs. controls (*p* = 0.05 uncorrected). **(B,C)** only include data from tasks which included both PWA and control data. Sample size denotes the number of tasks included in the ALE.

The language comprehension ALE included all 21 comprehension tasks with aphasia data. We also conducted this analysis using the 17 comprehension tasks that included PWA and control data; the coordinates associated with this analysis for PWA are reported in Supplementary Table 2 and depicted in Figure 4B. For all 21 comprehension tasks with aphasia data, the ALE identified nine significant clusters activated by PWA. Seven clusters were located in the left hemisphere with the largest peak being in the left middle frontal gyrus. Smaller peaks were found in the left inferior frontal gyrus (pars orbitalis), left medial frontal gyrus, left precentral gyrus, left posterior superior temporal gyrus, and left middle temporal gyrus (one anterior and one posterior peak). Additional peak activations in the right hemisphere were found in the right middle frontal gyrus and right claustrum (*p* < 0.001 corrected; Table 2; Figure 4A). We were not able to conduct *post-hoc* analyses for the aphasia subtypes as only two comprehension studies provided aphasia type-specific activation data (one for Broca's aphasia and one for Wernicke's aphasia). However, *post-hoc* analyses dividing the comprehension tasks by sensory modality (visual reading vs. auditory; Supplementary Table 5) indicate that these findings are likely driven by the auditory comprehension tasks because the peak activations for the auditory comprehension tasks are quite similar to the PWA comprehension ALE (Figure 4A; Table 2), and the visual comprehension tasks did not elicit any significant activations at the threshold of *p* < 0.001 corrected.

#### Controls

Seventeen comprehension tasks included control data. The control comprehension ALE identified three clusters to be significantly activated during comprehension tasks. The largest cluster's peak was in the left middle frontal gyrus. Two additional peaks were identified in the left inferior frontal gyrus (pars triangularis) and left middle temporal gyrus (*p* < 0.001 corrected; Supplementary Table 3; Figure 4B).

#### PWA vs. Controls

Seventeen comprehension tasks included PWA and control data. Four significant clusters were identified in both PWA and controls during language comprehension: three peaks were in the left inferior frontal gyrus (pars opercularis, pars triangularis, pars orbitalis) with the largest cluster being in the left pars orbitalis. One additional peak, in the left posterior middle temporal gyrus, was also observed to be activated by both PWA and controls during language comprehension. PWA significantly activated the left precentral gyrus more so than controls, while controls significantly activated the left middle frontal gyrus more than PWA (*p* = 0.05 uncorrected; Table 3; Figure 4C).

### Neural Resources Engaged by Language Production vs. Comprehension

To further explore the contributions of the right hemisphere to language in post-stroke aphasia, we contrasted activation for language production and comprehension in PWA and controls. Here we report the ALE findings for production greater than comprehension, comprehension greater than production, and their conjunction.

#### All PWA

**Figure 5:**
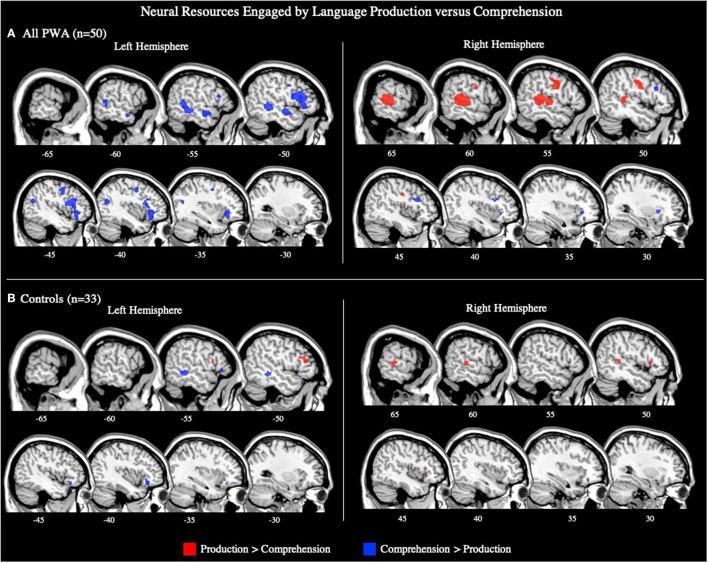
Representative sagittal slices for the production > comprehension and comprehension > production ALEs in **(A)** PWA and **(B)** controls (*p* = 0.05 uncorrected). Sample size denotes the number of tasks included in the ALE.

The ALE included data from all 29 production and 21 comprehension tasks (50 total) that reported aphasia data. The conjunction analysis identified seven significant clusters activated for language production and comprehension in PWA. These regions included the left middle, superior, and medial frontal gyri, left middle temporal gyrus (three clusters), and right anterior insula (largest cluster). The production greater than comprehension ALE in PWA identified the right precentral gyrus and right posterior superior temporal gyrus (largest cluster) to be more activated during language production than comprehension. For comprehension greater than production, PWA activated the left inferior frontal gyrus (pars triangularis) the most, but also the left superior frontal gyrus, left precentral gyrus, left posterior middle temporal gyrus (two peaks), left anterior superior temporal gyrus, right middle frontal gyrus, and right putamen (lentiform nucleus; *p* = 0.05 uncorrected; Table 2; Figure 5A). This same analysis was conducted using the 33 tasks (16 production, 17 comprehension) which included PWA and control data; the coordinates associated with this analysis for PWA are reported in Supplementary Table 2 and depicted in Supplementary Figure 2.

#### Controls

The ALE included data from the 16 production and 17 comprehension tasks (33 total) that included control data. The conjunction analysis identified eight clusters activated by production and comprehension in controls. These clusters had peaks in the left inferior frontal gyrus (pars orbitalis; largest cluster), left inferior frontal gyrus (pars triangularis; three peaks), left middle frontal gyrus (two peaks), left insula, and left middle temporal gyrus. The production greater than comprehension analysis identified five significant clusters. The largest cluster's peak was in the left medial frontal gyrus. Subsequent peaks were identified in the left middle frontal gyrus, right inferior frontal gyrus (pars triangularis), and right superior temporal gyrus (two peaks). Controls significantly activated two clusters for comprehension greater than production, the largest cluster was in the left middle temporal gyrus, and the other in the left inferior frontal gyrus (pars orbitalis) (*p* = 0.05 uncorrected; Supplementary Table 3; Figure 5B).

### The Effects of Task Type on the Neural Resources Engaged by Language^4^

#### Picture Naming Tasks

##### All PWA

**Figure 6:**
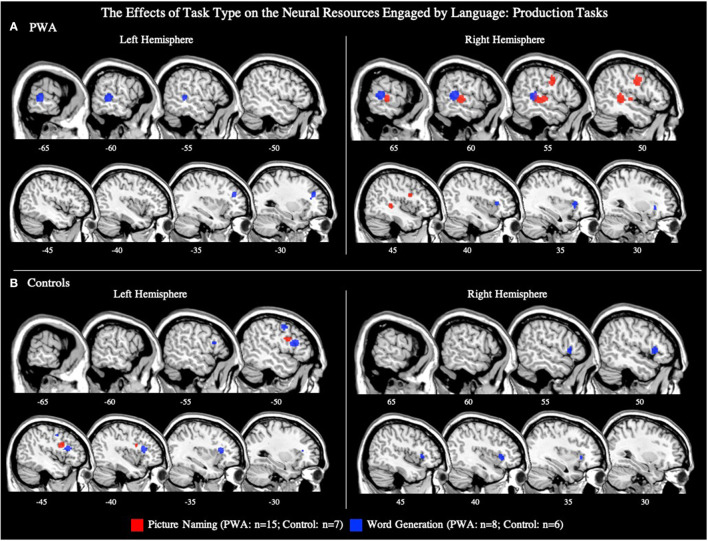
Representative sagittal slices for the picture naming and word generation ALEs in **(A)** PWA and **(B)** controls (*p* < 0.001 corrected). Sample size denotes the number of tasks included in the ALE.

Fifteen articles included a total of 106 PWA completing a picture naming task during scanning, for which an ALE was computed. We also conducted this analysis using the seven picture naming tasks that included both PWA and control data; the coordinates associated with this analysis for PWA are reported in **Table 5** and depicted in Supplementary Figure 3A (*p* < 0.001 corrected). The ALE using all 15 picture naming tasks identified two significant clusters in PWA. The largest cluster's peak was in the right posterior superior temporal gyrus and extended into the right posterior middle temporal gyrus. The smaller cluster's peak was in the right precentral gyrus and extended into the right inferior frontal gyrus (pars triangularis; *p* < 0.001 corrected; Table 4; Figure 6A).

**Table 4:** Anatomical locations of peak coordinates and cluster size for each single task ALE in PWA.

**Condition**	**Anatomical location**	**Peak coordinates**	**Voxels (mm^**3**^)**
Picture naming	Right precentral gyrus^*^, right inferior frontal gyrus (pars triangularis)	52, −2, 32	2,280
	Right superior temporal gyrus^*^, right middle temporal gyrus	52, −32, 6	4,040
Picture naming: Correct responses	Right precentral gyrus^*^	52, −2, 34	1,128
	Right superior temporal gyrus^*^, right middle temporal gyrus	62, −22, 2	1,816
Picture naming: Incorrect responses	Right inferior frontal gyrus (pars opercularis^*^)	50, 10, 22	8,688
	Right middle temporal gyrus^*^	50, −28, −2	1,416
Picture naming: Incorrect > Correct responses	Right inferior frontal gyrus (pars triangularis^*^), right insula, right middle frontal gyrus	46, 16, 18	4,680
Word generation	Left middle frontal gyrus^*^	−32, 38, 28	1,192
	Left cingulate gyrus^*^, right cingulate gyrus	−8, 12, 38	1,536
	Left middle temporal gyrus^*^	−62, −36, 4	1,904
	Right insula^*^, right inferior frontal gyrus (pars triangularis)	32, 24, −2	1,040
	Right superior temporal gyrus^*^	60, −34, 8	2,656
Semantic decision	Left inferior frontal gyrus (pars triangularis)^*^	−46, 24, 14	752
	Left middle temporal gyrus^*^	−42, −64, 22	848
Auditory sentence listening	Left inferior frontal gyrus (pars orbitalis)^*^	−40, 28, −4	2,128
	Left middle frontal gyrus^*^	−46, 20, 20	1,192
	Left superior frontal gyrus^*^	−8, 8, 52	968
	Left middle temporal gyrus^*^, left superior temporal gyrus	−50, −38, −2	2,224
	Left superior temporal gyrus^*^, left inferior temporal gyrus, left middle temporal gyrus	−52, −8, −8	2,096
	Right claustrum^*^	30, 20, 4	896

*The x, y, z coordinates are in Talairach space and refer to the peak voxel activated in each cluster. All single condition ALEs are thresholded at p < 0.001 corrected and contrast ALEs at p = 0.05 uncorrected. Asterisks indicate anatomical location of peak voxel*.

**Figure 7:**
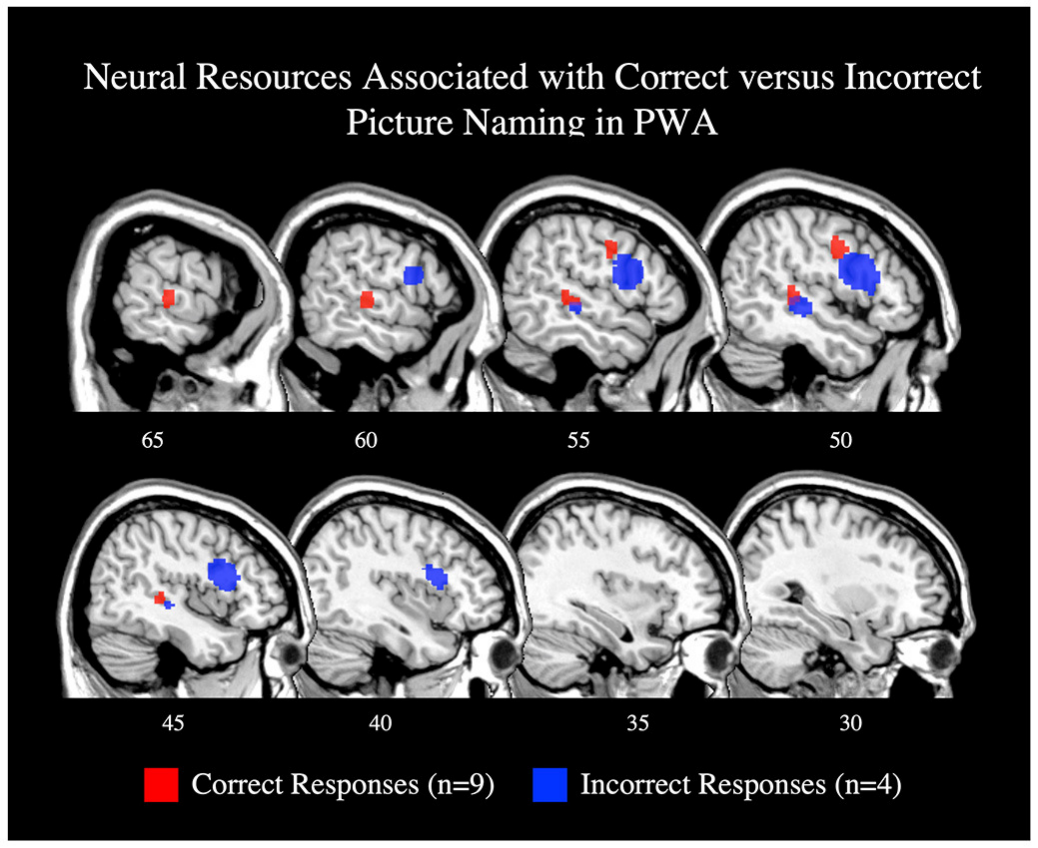
Representative sagittal slices for the ALEs associating picture naming activation with correct responses (red) and incorrect responses (blue; *p* < 0.001 corrected). Only the right hemisphere is depicted. Sample size denotes the number of tasks included in the ALE.

To explore activations related to naming task performance, we additionally conducted *post-hoc* ALEs to explore whether picture naming activation patterns differed for correctly and incorrectly named items. The ALE for correct responses included nine studies with 78 PWA. This ALE identified two significant clusters with peaks in the right posterior superior temporal gyrus and right precentral gyrus; this finding aligns with the results of the main picture naming ALE (Table 4; Figure 7; *p* < 0.001 corrected). The ALE for incorrectly named items should be interpreted with caution as it only included four studies with 12 PWA. Nonetheless, this ALE did identify activation of voxels in the right inferior frontal gyrus (pars opercularis) and right posterior middle temporal gyrus when items were incorrectly named (Table 4; Figure 7; *p* < 0.001 corrected). No brain regions were significantly more active during correct naming than incorrect naming, but voxels in the right inferior frontal gyrus (pars triangularis) were found to be significantly more activated during incorrect naming than correct naming (Table 4; *p* = 0.05 uncorrected).

##### Controls

Seven articles included 105 control participants completing a picture naming task during scanning. The control ALE identified one cluster in the left inferior frontal gyrus (pars triangularis; *p* < 0.001 corrected; Table 5; Figure 6B).

**Table 5:** Anatomical locations of peak coordinates and cluster size for each single condition ALE in PWA and controls, as well as contrast ALEs comparing PWA and controls on each individual language task.

**Condition**	**Anatomical location**	**Peak coordinates**	**Voxels (mm^**3**^)**
**Picture naming**			
PWA	Right superior temporal gyrus^*^	52, −32, 8	720
	Right superior temporal gyrus^*^	60, −22, 0	632
Controls	Left inferior frontal gyrus (pars triangularis^*^)	−46, 8, 24	1,296
Controls > PWA	Left inferior frontal gyrus (pars triangularis^*^, pars opercularis)	−48.2, 5.5, 26.3	1,904
	Left middle frontal gyrus^*^	−50, 28, 22	1,136
	Left medial frontal gyrus^*^	−2.7, 2.7, 56	488
	Right superior temporal gyrus^*^	50, 12, −6	312
**Word generation**			
PWA	Right insula^*^, right inferior frontal gyrus (pars triangularis)	32, 24, −2	1,240
	Right middle temporal gyrus^*^	58, −36, 6	712
Controls	Left inferior frontal gyrus (pars triangularis^*^), left insula	−50, 18, 18	3,232
	Left superior frontal gyrus^*^, right superior frontal gyrus	−2, 18, 48	2,000
	Left precentral gyrus^*^	−48, 0, 46	696
	Right inferior frontal gyrus (pars triangularis^*^, pars orbitalis)	52, 20, 6	1,840
Conjunction	Right inferior frontal gyrus (pars orbitalis^*^)	36, 24, 2	168
Controls > PWA	Left inferior frontal gyrus (pars triangularis)^*^	−46, 18, 14	2,264
	Left middle frontal gyrus^*^, left precentral gyrus	−47, 1, 42	696
	Left superior frontal gyrus^*^	−6.8, 15.1, 52.9	1,968
	Right inferior frontal gyrus (pars triangularis^*^)	48, 22, 0	1,000
**Semantic decision**			
PWA	Left inferior frontal gyrus (pars triangularis^*^)	−46, 24, 14	808
	Left middle temporal gyrus^*^	−42, −64, 22	968
	Left middle temporal gyrus^*^	−56, −32, −10	624
**Auditory sentence listening**			
PWA	Left inferior frontal gyrus (pars orbitalis^*^)	−40, 28, −4	2,144
	Left middle frontal gyrus^*^	−46, 20, 20	1,192
	Left superior frontal gyrus^*^	−8, 8, 52	968
	Left middle temporal gyrus^*^, left superior temporal gyrus	−50, −38, −2	2,240
	Left superior temporal gyrus^*^, left inferior temporal gyrus, left middle temporal gyrus	−52, −8, −8	2,112
	Right claustrum^*^	30, 20, 4	896
Controls	Left middle temporal gyrus^*^	−56, −42, 2	1,448
Conjunction	Left middle temporal gyrus^*^	−56, −42, 4	1,176

*Only studies which included both PWA and control data are included in these analyses*.

*The x, y, z coordinates are in Talairach space and refer to the peak voxel activated in each cluster. All contrast ALEs are thresholded at p = 0.05 uncorrected. Asterisks indicate anatomical location of peak voxel*.

##### PWA vs. Controls

Seven articles included 89 PWA and 105 control participants completing the same picture naming task during scanning. The conjunction ALE identified no significant clusters to be activated by both PWA and controls. PWA did not activate any brain region more than controls. However, the control greater than PWA ALE identified significant peaks in the left inferior frontal gyrus (pars orbitalis; largest cluster), left middle frontal gyrus, left medial frontal gyrus, and right superior temporal gyrus (*p* = 0.05 uncorrected; Table 5).

#### Word Generation Tasks

##### All PWA

Eight articles included word generation tasks while scanning 62 PWA. The ALE for word generation resulted in five significant clusters with the largest peak being in the right posterior superior temporal gyrus, and smaller peaks in the right anterior insula, left posterior middle temporal gyrus, left cingulate gyrus, and left middle frontal gyrus (*p* < 0.001 corrected; Table 4; Figure 6A). We also conducted this analysis using just the six word generation tasks that included PWA and control data; the coordinates associated with this analysis for PWA are reported in Table 5 and depicted in Supplementary Figure 3A (*p* < 0.001 corrected). A *post-hoc* ALE of performance-related activations for word generation tasks could not be computed because no word generation study reported whether the coordinates were associated with correct or incorrect responses.

##### Controls

Six articles included 63 control participants completing a word generation task during scanning. The control ALE identified four significant clusters with peaks in the left inferior frontal gyrus (pars triangularis; largest cluster), left superior frontal gyrus, left precentral gyrus, and right inferior frontal gyrus (pars triangularis, pars orbitalis; *p* < 0.001 corrected; Table 5; Figure 6B).

##### PWA vs. Controls

Six articles included 43 PWA and 63 control participants completing the same word generation task. PWA and controls both activated the right inferior frontal gyrus (pars orbitalis) during word generation. PWA activated no brain regions more than controls. The control greater than PWA ALE identified four significant clusters including the left inferior frontal gyrus (pars triangularis; largest cluster), left middle frontal gyrus, left superior frontal gyrus, and right inferior frontal gyrus (pars triangularis; *p* = 0.05 uncorrected; Table 5).

#### Semantic Decision Tasks

##### All PWA

**Figure 8:**
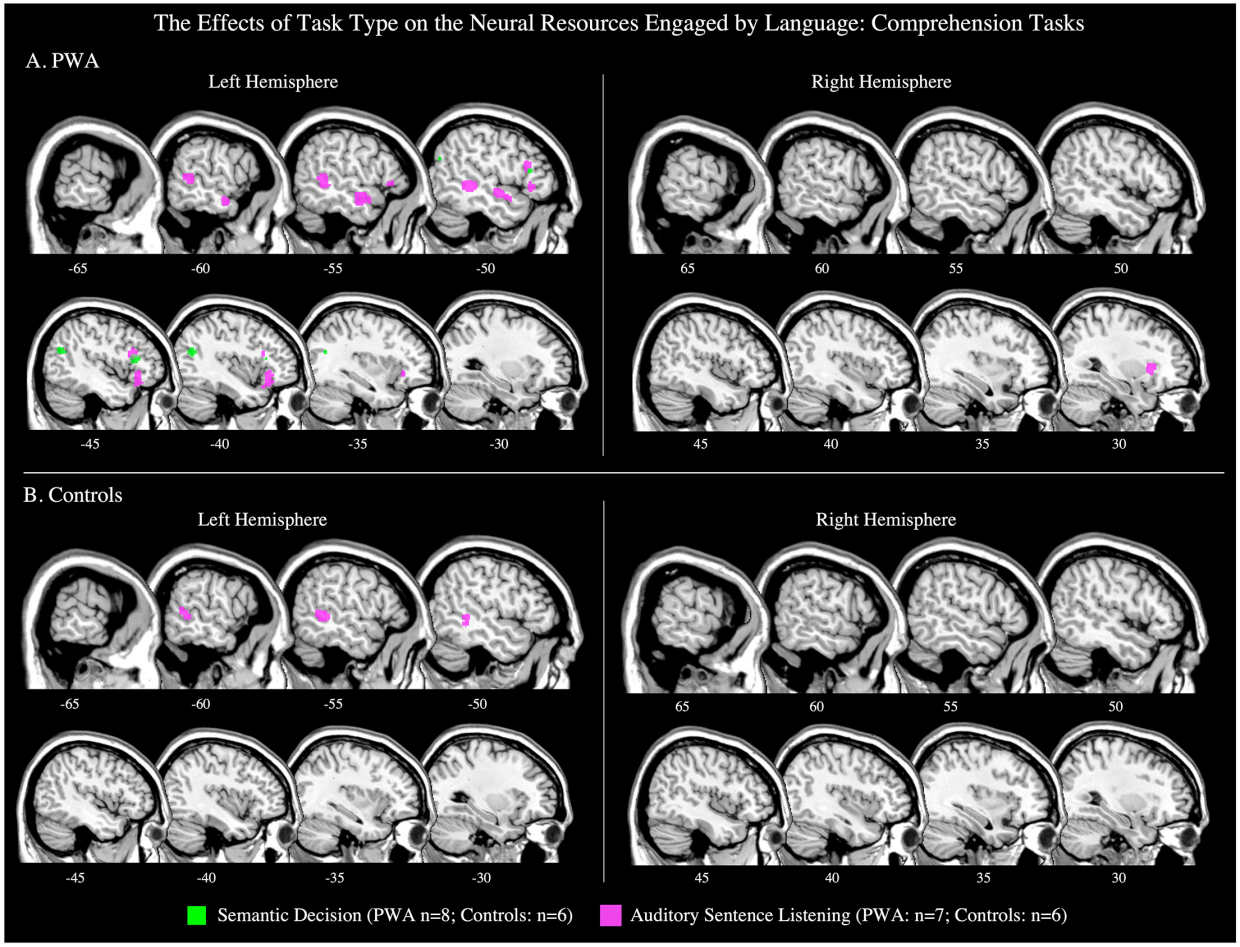
Representative sagittal slices for the semantic decision and auditory sentence listening ALEs in **(A)** PWA and **(B)** controls (*p* < 0.001 corrected). Sample size denotes the number of tasks included in the ALE.

Eight articles included 91 PWA completing a semantic decision task during scanning. The ALE for semantic decisions identified two significant clusters; the largest cluster peaked in the left posterior middle temporal gyrus and the smaller peak was in the left inferior frontal gyrus (pars triangularis; *p* < 0.001 corrected; Table 4; Figure 8A). We also conducted this analysis using just the six semantic decision tasks that included PWA and control data; the coordinates associated with this analysis are reported in Table 5 and depicted in Supplementary Figure 3B (*p* < 0.001 corrected). A *post-hoc* ALE of performance-related activations for semantic decision tasks could not be computed because only two studies reported whether the coordinates were associated with correct or incorrect responses.

##### Controls

Six articles included 70 control participants completing a semantic decision task during scanning. The control ALE identified no significant clusters (*p* < 0.001 corrected; Table 5; Figure 8B); because of this the contrast comparing PWA and controls could not be computed.

#### Auditory Sentence Listening Tasks

##### All PWA

In seven studies, 109 PWA listened to auditory sentences during scanning. The ALE for auditory sentence listening identified six clusters. Five clusters were located in the left hemisphere: the largest peak was in the left inferior frontal gyrus (pars orbitalis), and smaller peaks were identified in the left middle frontal, superior frontal, posterior middle temporal, and anterior superior temporal gyri. The sixth peak was in the right claustrum (*p* < 0.001 corrected; Table 4; Figure 8A). We also conducted this analysis using just the six sentence listening studies that included PWA and control data; the coordinates associated with this analysis for PWA are reported in Table 5 and depicted in Supplementary Figure 3B (*p* < 0.001 corrected). A *post-hoc* ALE of performance-related activations for auditory sentence listening tasks could not be computed because no study reported whether the coordinates were associated with correct or incorrect responses.

##### Controls

Six articles included 91 control participants completing an auditory listening task during scanning. The control ALE identified one significant cluster in the left middle temporal gyrus (*p* < 0.001 corrected; Table 5; Figure 8B).

##### PWA vs. Controls

Six articles included 108 PWA and 91 control participants completing the same auditory listening task during scanning. The ALE conjunction analysis identified PWA and controls to both activate the left middle temporal gyrus, however, neither PWA nor controls activated any brain region more than the other group (*p* = 0.05 uncorrected; Table 5).

## Discussion

The present meta-analysis investigated how the left and right hemispheres are engaged during language production and comprehension in persons with chronic aphasia. We further sought to characterize how the neural resources engaged by language production and comprehension in PWA compares to what is observed in control subjects, as well as how the neural resources may differ based on task type. As expected, we found that PWA activated bilateral frontal and temporal cortices during language production and comprehension, similar to controls. However, unlike what has been previously reported in controls (e.g., LaCroix et al., 2015; Rodd et al., 2015), we find PWA to demonstrate greater overall activation of the right hemisphere for language production compared to comprehension, while the left hemisphere exhibited greater activation for comprehension than production. The implications of our findings regarding the functional reorganization of language in post-stroke aphasia, and how they may be related to the recruitment of domain-general cognitive resources are discussed below.

### Neural Resources Engaged by Language in PWA vs. Controls

As expected, our combined analysis investigating the neural resources engaged by language in PWA, across all production and comprehension tasks, identified a bilateral fronto-temporal network, coinciding with dual-stream models of speech processing (e.g., Hickok and Poeppel, 2007; Friederici, 2011; Bornkessel-Schlesewsky and Schlesewsky, 2013). Specifically, PWA activated the canonical language network including the left middle frontal and temporal gyri, as well as left perilesional (e.g., tissue adjacent to the canonical language network) and right hemisphere regions (e.g., right insula). These findings align with a previous meta-analysis, in which PWA activated spared regions within the left lateralized language network, plus left perilesional and right hemisphere homologs including the right inferior frontal gyrus (pars orbitalis), right middle frontal gyrus, right insula, and right middle temporal gyrus (Turkeltaub et al., 2011). This same analysis in our control group revealed a similar bilateral fronto-temporal network, with additional activations being observed in the left superior and inferior parietal lobes. We further found that both PWA and controls activate left frontal regions (e.g., left inferior frontal gyrus) and bilateral superior and middle temporal gyri in response to all language tasks; this activation pattern was expected based on the dual stream models. In addition, PWA and controls both engaged bilateral regions implicated in multiple cognitive-linguistic functions, including the bilateral superior and medial frontal gyri and insula, across all language tasks.

While the current and previous meta-analyses cannot fully account for behavioral performance, previous work associates left hemisphere activation (see Wilson and Schneck, 2021 for a review), and to a lesser extent, activation of the right middle temporal gyrus with better language outcomes in PWA (Price and Crinion, 2005; Tyler et al., 2011). Resting-state fMRI studies have also found increased functional connectivity within the left hemisphere to be associated with greater language abilities (Siegel et al., 2016). These previous results, in addition to our ALE contrast indicating that only a small cluster in the right inferior frontal gyrus (pars triangularis) was more activated in PWA than controls across all language tasks, suggest that spared regions within and adjacent to the canonical language network, as well as more domain-general regions typically activated in controls are engaged during language tasks in PWA (Price and Crinion, 2005; Crinion et al., 2006; Warren et al., 2009; Fridriksson et al., 2010; Tyler et al., 2011; Allendorfer et al., 2012; Robson et al., 2014; Griffis et al., 2017; Nenert et al., 2018). Although lesion volume could not be accounted for in our study, previous work indicates that increased activation of the right hemisphere results in better language outcomes, particularly when large portions of the left hemisphere are lesioned (Karbe et al., 1998; Cao et al., 1999; Blasi et al., 2002; Heiss and Thiel, 2006; Sebastian and Kiran, 2011; Griffis et al., 2017; Skipper-Kallal et al., 2017). Thus, right hemisphere activations in PWA, such as in the right inferior frontal gyrus, may reflect compensation for widespread damage to portions of the left lateralized canonical language network.

### Language Production Engages the Right Hemisphere in PWA

Bilateral fronto-temporal cortices were activated by PWA during language production tasks. The ALE in control subjects indicated a similar network, but more left lateralized. Although the direct contrast of PWA vs. controls indicates that PWA did not activate any regions more than controls, the within-group ALEs depict large swaths of activation in right motor and pre-motor cortices in PWA, but much smaller right hemisphere frontal activations in controls. This discrepancy in findings could be because only half of the production studies included control data, so the between-group contrasts had reduced power compared to the within-group comparisons. The bilateral organization of the neural resources engaged by production in PWA was somewhat unexpected as it is well-established that language production is highly left lateralized (e.g., Hickok and Poeppel, 2007; Friederici, 2011; Bornkessel-Schlesewsky and Schlesewsky, 2013). To gain more insight into the mechanisms that may be driving right hemisphere engagement during language production in post-stroke aphasia, we examined not only the brain regions activated during language production, but also those activated more for production than comprehension (section Neural Resources Engaged by Language Production vs. Comprehension). We posit that regions significantly activated in both analyses may reflect involvement in computations specific to speech production, as opposed to domain-general functions (e.g., general alertness or effort) or language production or comprehension processes. To this end, we identified the right precentral gyrus and the right superior temporal gyrus to be significantly activated in both analyses in PWA; no left hemisphere regions were identified. It is possible that these regions are compensating for damage to regions which support motor speech planning for language, but certainly further work is needed to determine the specificity of these activations. This same analysis in controls identified a similar, but bilateral activation pattern: controls activated the right superior temporal gyrus and the right inferior frontal gyrus, but also, as expected based on previous work, left inferior, middle, and superior frontal gyri. The shared right superior temporal gyrus activation for PWA and controls is likely associated with processing one's own speech (Yamamoto et al., 2019) as the majority of language production tasks (22/29) required an overt response. The right frontal activations are likely tied to articulatory processes (Price, 2012). The overlap in findings in the right hemisphere for PWA and controls indicates that PWA likely engage right hemisphere resources already involved in language production to compensate for damage to the canonical language network, and that these right hemisphere resources appear to be involved in functions specific to speech production, rather than more domain-general functions.

Our ALE exploring the effect of task on language production activations suggests that not all right hemisphere activation during language production in PWA is tied to language-specific processes. Instead, the production task sub-analysis suggests that some right hemisphere activation, particularly in the frontal lobes, may depend on cognitive demands inherent to the task. For example, the lexical retrieval aspects of picture naming have been associated with the left anterior and posterior temporal cortex (Damasio et al., 2004; Schwartz et al., 2009; Walker et al., 2011; Baldo et al., 2013), and the articulatory planning and programming components with frontal regions, including the left inferior frontal gyrus (DeLeon et al., 2007; though others implicate this region with semantic processing; e.g., Price, 2012). Though underpowered with seven articles, the controls' picture naming ALE associated the left inferior frontal gyrus (pars triangularis) with picture naming. The PWA's picture naming ALE was adequately powered with 15 studies and identified a similar pattern of activation to controls, but in the right hemisphere: PWA activated the right precentral gyrus and right superior temporal gyrus. Notably, this right hemisphere activation pattern in PWA during naming tasks appears to be driven by correct responses, coinciding with previous studies indicating that increased activation of the right precentral gyrus is associated with improved naming abilities in post-stroke aphasia (e.g., Fridriksson et al., 2009; Postman-Caucheteux et al., 2010; Skipper-Kallal et al., 2017).

In contrast to picture naming tasks, word generation tasks additionally require executive functions to select words that meet task constraints (e.g., naming words that begin with the letter “M” without including proper nouns or repeating the same word with a different ending; Amunts et al., 2020). During word generation tasks, we find PWA to activate the left middle frontal gyrus, right inferior frontal gyrus (pars triangularis), and bilateral cingulate gyri, all of which have been associated with executive functions in controls in the present study, but also in past work (e.g., Schlosser et al., 1998; Fu et al., 2002; Costafreda et al., 2006; Nagels et al., 2012; Marsolais et al., 2015). The inherent differences in the use of executive functions for word generation vs. picture naming likely explains the more bilateral frontal activation PWA and controls demonstrate during word generation tasks compared to picture naming tasks. Thus, these task-specific ALEs during language production suggest that motor speech planning for language production is supported by right hemisphere homologs in post-stroke aphasia, while activations beyond these regions, in either hemisphere, are likely driven to some extent by more domain-general cognitive functions.

The relationship between behavioral performance and neural activation was sparsely reported in the studies that were possible to include in the present meta-analysis—see the limitations section below for more discussion. However, we did compute ALEs for task-related activations when possible, i.e., for correct and incorrect picture naming responses (although these findings should be interpreted with caution given the small sample size, i.e., nine and four studies, respectively). Correct and incorrect responses both activated right fronto-temporal regions: correct responses activated the right precentral gyrus and right superior temporal gyrus, and incorrect responses activated the right inferior frontal gyrus (pars opercularis) and right middle temporal gyrus. Contrasting correct and incorrect responses further linked the right inferior frontal gyrus (pars triangularis) with unsuccessful naming. Although activation of the right inferior frontal gyrus has generally been associated with better language abilities, particularly when the left inferior frontal gyrus is lesioned (Fridriksson et al., 2009; Sebastian and Kiran, 2011; Turkeltaub et al., 2011; Harvey et al., 2017; Skipper-Kallal et al., 2017), it seems that the right pars triangularis and pars opercularis should be examined separately as previous work indicates that the right pars opercularis functions similarly to the left pars opercularis, but that the right pars triangularis functions differently than the left pars triangularis (Turkeltaub et al., 2011). Together, our results (and others) show that the right hemisphere has a multifaceted contribution to spoken language production, but that activation of the right pars triangularis appears to be particularly detrimental to language recovery in post-stroke aphasia (Naeser et al., 2011; Turkeltaub et al., 2011; Harvey et al., 2017). Future work using correlation or regression analyses are needed to better identify activations which significantly predict successful language production abilities in PWA.

### Language Comprehension Engages the Left Hemisphere in PWA

During language comprehension tasks, PWA and controls activated several left hemisphere regions including large clusters in the posterior and anterior middle and superior temporal gyri and left inferior frontal gyrus—coinciding with dual-stream models. However, findings in the right hemisphere deviated from our expectations based on previous findings of bilateral temporal activations during language comprehension: in the right hemisphere, PWA activated the right middle frontal gyrus and right claustrum but no right temporal regions, and controls did not significantly activate any right hemisphere regions. The left ventral stream activation in PWA and controls coincides with previous lesion-symptom mapping studies indicating that left temporal cortices are critical to single word (Bates et al., 2003; Newhart et al., 2007; Bonilha et al., 2017) and sentence comprehension in PWA (Dronkers et al., 2004; Thothathiri et al., 2012; Magnusdottir et al., 2013; Pillay et al., 2017; Rogalsky et al., 2018). However, dual stream models (e.g., Hickok and Poeppel, 2007; Friederici, 2011; Bornkessel-Schlesewsky and Schlesewsky, 2013) and fMRI/PET studies in controls (e.g., LaCroix et al., 2015; Rodd et al., 2015) also reliably implicate the right hemisphere in receptive language tasks, so the lack of right temporal activations identified by the comprehension ALEs was surprising. The lack of right temporal lobe findings for comprehension may be an artifact of the types of tasks used in the studies meeting our inclusion criteria, i.e., approximately one-third were sentence-level tasks and one-third were semantic decision tasks, and both sentence-level comprehension and lexical-semantic processes are left-dominant (Hickok and Poeppel, 2007; Friederici, 2011; Bornkessel-Schlesewsky and Schlesewsky, 2013; Rogalsky et al., 2018). Thus, it is possible that the more consistent involvement of the left hemisphere for language comprehension in our study (in PWA and controls) may be driven to some extent by stimulus type. The ALEs exploring the effect of task type on the neural resources engaged by language comprehension lend some additional support to this possibility as the auditory sentence listening ALE results highly overlap with the overall language comprehension ALE, particularly in PWA.

Cognitive processes associated with task completion may explain recruitment of the left frontal cortex during language comprehension in PWA and controls. PWA and controls both activated the left inferior and middle frontal gyri during the comprehension tasks, as well as the left precentral gyrus, left middle temporal gyrus, and right claustrum. While it is unclear from the present study whether engagement of domain-general resources improves behavioral performance or not, the relationship between frontal regions and domain-general computations is well-established (e.g., Ries et al., 2016; Fedorenko and Blank, 2020), as is the relationship between the claustrum and attention (e.g., Crick and Koch, 2005; Mathur, 2014; Goll et al., 2015; Smith et al., 2020). There is also a small body of work that links the left middle temporal gyrus with working memory (e.g., Gläscher et al., 2009), however, it is more commonly linked to passive listening (e.g., Crinion and Price, 2005; Rogalsky et al., 2018). Notably, out of all these shared activations, only the left precentral gyrus was more activated in PWA compared to controls. This increased activation of the left precentral gyrus, in a region of motor cortex closer to the hand than mouth area, is likely due to increased effort of PWA in their motor responses, not domain-general cognitive processes, as 18 of the 21 studies included in the comprehension ALE required participants to make an overt judgment about the stimulus, typically through a button press. The only region more activated by controls than PWA during comprehension tasks was a small cluster in the left inferior frontal gyrus (pars opercularis), an area that PWA also reliably activated. This finding suggests that non-lesioned frontal resources may play some role in language comprehension in post-stroke aphasia, but that their recruitment is not unique to PWA, and therefore may not be compensatory. However, previous work does propose that activation of domain-general resources may upregulate the remaining intact portions of the canonical language network (Diachek et al., 2020). Thus, future studies are needed to better characterize the contributions of domain-general cognitive resources to the functional reorganization of language functions in post-stroke aphasia.

### Limitations and Future Directions

While the ALE methodology has several strengths, which allow it to overcome some of the limitations of individual studies (e.g., small sample size, inadequate power, differences in task), there are nonetheless limitations. First, our inclusion criteria were limited to PWA in the chronic recovery stage. Thus, our findings cannot be extended to the acute and sub-acute phases. In the acute and sub-acute phases, the current evidence indicates that language is initially supported by right hemisphere resources before gradually transitioning to left perilesional regions as language recovers (Saur et al., 2006; Nenert et al., 2018; Hartwigsen and Saur, 2019). For example, Saur et al. (2006) found auditory comprehension performance to be positively correlated with acute right inferior frontal gyrus activation, but in the chronic stage, left hemisphere activation was associated with better comprehension. While a meta-analysis of acute and sub-acute language recovery in post-stroke aphasia is beyond the scope of this paper, it would certainly further our understanding of the trajectory of functional activation differences in post-stroke language recovery.

This meta-analysis is also limited in that it only partially accounts for the relationship between neural activation and behavioral performance. This is not due to a methodological limitation of the current study, but rather a function of what studies are available in the published literature for us to input into our meta-analysis. While there was (marginally) sufficient data to conduct *post-hoc* ALEs that examine activations related to correctly and incorrectly named items during picture naming, we were not able to do the same for the other production or comprehension tasks due to the multitude of ways in which performance (if reported) was described in each study (e.g., coordinates, correlations, text descriptions, figures). In total, 11/50 tasks reported coordinates associated with behavioral performance of some kind. Of these 11 studies, nine were the picture naming tasks we computed ALEs for, and two were comprehension tasks (both semantic decision tasks). To overcome this limitation, future fMRI and PET studies of language recovery in aphasia should ideally include correlations between behavioral performance and brain activations, and separately report coordinates for correct and incorrect responses. This type of consistency in the literature will strengthen our understanding of the effectiveness of compensatory strategies to post-stroke language recovery.

Our inclusion criteria were further limited to whole brain analyses and tasks that utilized a non-speech baseline task (e.g., listening to tones, rest). While each of these criteria was necessary to balance sufficient power and homogeneity (Müller et al., 2018), it did result in the exclusion of several articles (Figure 1; Supplementary Table 1). However, with these criteria, we still identified a sufficient number of studies to achieve appropriate power for our main analysis of interest (Eickhoff et al., 2016): how the neural resources engaged by language production vs. comprehension differ in PWA. Nonetheless, we excluded 12 studies which did not report a whole-brain analysis (e.g., ROI, VOI) and 16 studies that contrasted a language task with a language baseline (e.g., spontaneous speech vs. repeated speech). We also excluded 51 studies that did not report any, or only partial, functional activation coordinates. This heterogeneity within functional neuroimaging studies of aphasia recovery suggests a need for general fMRI and PET reporting guidelines. We suggest, that at a minimum, functional imaging studies of language recovery in post-stroke aphasia should report coordinates from whole-brain analyses where the primary task of interest is compared to rest, in addition to their primary analyses of interest, with the caveats noted that rest does not adequately control for non-language related activations and may actually subtract activation related to semantic processing from semantic decision tasks (Binder et al., 2009). We also second recommendations made by Wilson and Schneck (2021) regarding mechanisms to reduce task performance confounds and improve contrast validity. The adoption of general result reporting guidelines will reduce bias within the field as there will be greater homogeneity across studies for future meta- and mega-analyses of functional imaging studies, which will be instrumental in furthering our understanding of language recovery in post-stroke aphasia.

A final limitation of the present work is our focus on studies reporting coordinates not related to treatment. Thus, from the present meta-analysis, it is difficult to infer how treatment affects the neural resources supporting language. The heterogeneity of treatment interventions and methodological approaches makes it difficult to include these studies in an ALE meta-analysis, yet there is certainly evidence that behavioral treatments can influence the neural resources that are recruited during language tasks. For example, Cherney and Small (2006) report on a case involving a PWA with a left frontal and anterior temporal lesion. Prior to treatment, the PWA had no activation in either hemisphere during an oral reading task, however, following treatment, activation of right hemisphere homologs was observed. These results further indicate that the right hemisphere appears better able to support language production when the left language network is damaged, and that this recruitment may be driven by treatment. Other studies show similar changes in the neural resources supporting language following treatment (Musso et al., 1999; Meinzer et al., 2008; Raboyeau et al., 2008; Marcotte et al., 2012; Tabei et al., 2016). Since it is well-established that language can continue to improve following therapy administered in the chronic stage (e.g., Brady et al., 2016), there is a continued need to investigate how therapy impacts the neural resources supporting language recovery. This is particularly important since therapy-induced changes in the brain likely impact all neuroimaging studies of language recovery since most PWA receive some treatment in the acute and/or chronic stages that is not accounted for in many fMRI studies. Thus, continued work is needed to investigate likely differences in the brain regions supporting spontaneous language recovery and those that may be recruited secondary to treatment in order to better understand each process separately, as well as their interaction.

## Conclusion

Our exhaustive meta-analysis of language activations in PWA identified different intra- and inter-hemispheric functional organization patterns for production and comprehension. As expected, PWA activated the left middle and superior temporal gyri during language comprehension. We found additional intra-hemispheric activations in the left frontal gyri, but notably none in the right temporal lobes, which contrasted with what was expected based on predictions from the dual stream models (Hickok and Poeppel, 2007; Friederici, 2011; Bornkessel-Schlesewsky and Schlesewsky, 2013). Further analyses suggest that the left lateralized perilesional engagement during comprehension may be driven by a combination of stimulus complexity and recruitment of domain-general cognitive resources. In contrast to the comprehension results, production was associated with activation of the right frontal and temporal cortices in PWA. We also found that activation of regions known to support domain-general resources, such as the right inferior frontal gyrus, particularly the pars triangularis, were associated with unsuccessful naming, while activation of regions involved in motor speech planning for language production, such as the right precentral gyrus, were linked to successful naming. Overall, the within-group findings indicate that the neural resources engaged by language in post-stroke aphasia, and the engagement of the right hemisphere, differ for expressive and receptive language processes, with more right hemisphere involvement seen for production than comprehension. However, the overall similarities in areas activated by PWA and controls indicates that PWA likely engage similar neural resources during language tasks as controls, rather than recruiting unique resources.

## Data Availability Statement

The original contributions presented in the study are included in the article/Supplementary Material, further inquiries can be directed to the corresponding author/s.

## Author Contributions

AL and CR contributed to study conception and design. AL and EJ conducted the literature search and performed the statistical analyses. AL wrote the first draft of the manuscript. All authors contributed to manuscript revision, read, and approved the submitted version.

## Funding

This research was supported by Midwestern University and Arizona State University.

## Conflict of Interest

The authors declare that the research was conducted in the absence of any commercial or financial relationships that could be construed as a potential conflict of interest.

## Publisher's Note

All claims expressed in this article are solely those of the authors and do not necessarily represent those of their affiliated organizations, or those of the publisher, the editors and the reviewers. Any product that may be evaluated in this article, or claim that may be made by its manufacturer, is not guaranteed or endorsed by the publisher.

## Footnotes

^1^For the purposes of this paper, we define the canonical language network as the left inferior frontal and precentral gyri, left insula, left temporal-parietal junction, left anterior temporal lobe (middle and inferior temporal gyri), bilateral mid superior temporal gyri, and bilateral mid-posterior middle temporal gyri (Hickok and Poeppel, 2007; Friederici, 2011; Price, 2012; Bornkessel-Schlesewsky and Schlesewsky, 2013).

^2^“Perilesional tissue” and “perilesional activations” are routinely used in the extant literature to describe fMRI/PET activations which are adjacent to the area defined as lesioned in studies with access to a PWA's exact lesion location. However, the term perilesional is also used in meta-analyses similar to the present study (e.g., Turkeltaub et al., 2011). In these instances, perilesional refers to the residual tissue surrounding the lesion which is activated, and thus suggests that the tissue is not lesioned in the majority of participants included in the analysis. Therefore, we will use the term perilesional to discuss the results of this paper in accordance with this definition.

^3^Our meta-analysis search terms were quite similar, but not identical to Wilson and Schneck's (2021). This slight difference in search terms likely explains why these eight articles were not originally identified in our PubMed or Google Scholar searches.
