# Supplementary material for: Neural Resources Supporting Language Production vs. Comprehension in Chronic Post-stroke Aphasia: A Meta-Analysis Using Activation Likelihood Estimates
Source: Front Hum Neurosci. 2021 Oct 25;15:680933. doi: 10.3389/fnhum.2021.680933 (PMC8572938; doi:10.3389/fnhum.2021.680933)
Supplement: Supplementary file 1 [file Data_Sheet_1.docx]

Supplementary Table 1. Articles excluded from the meta-analysis organized by disqualifying criteria. Note, several studies did not meet multiple criteria. In these cases, the citation is listed under the primary disqualifier. Excluded articles which report production and comprehension tasks are listed once under “Production” with an asterisk.

| **Production (82 excluded)** | **Comprehension (55 excluded; 2 duplicates w/ production)** |
| --- | --- |
| **No Coordinates Reported** | |
| 1. Hillis, A. E., Kleinman, J. T., Newhart, M., Heidler-Gary, J., Gottesman, R., Barker, P. B., ... & Chaudhry, P. (2006). Restoring cerebral blood flow reveals neural regions critical for naming. The Journal of Neuroscience, 26(31), 8069-8073. 2. Winhuisen, L., Thiel, A., Schumacher, B., Kessler, J., Rudolf, J., Haupt, W. F., & Heiss, W. D. (2007). The right inferior frontal gyrus and poststroke aphasia a follow-up investigation. Stroke, 38(4), 1286-1292. 3. Kessler, J., Thiel, A., Karbe, H., & Heiss, W. D. (2000). Piracetam improves activated blood flow and facilitates rehabilitation of poststroke aphasic patients. Stroke, 31(9), 2112-2116. 4. Heiss, W. D., Kessler, J., Thiel, A., Ghaemi, M., & Karbe, H. (1999). Differential capacity of left and right hemispheric areas for compensation of poststroke aphasia. Annals of neurology, 45(4), 430-438. 5. Ohyama, M., Senda, M., Kitamura, S., Ishii, K., Mishina, M., & Terashi, A. (1996). Role of the Nondominant Hemisphere and Undamaged Area During Word Repetition in Poststroke Aphasics A PET Activation Study. Stroke, 27(5), 897-903. 6. Karbe, H., Thiel, A., Weber-Luxenburger, G., Herholz, K., Kessler, J., & Heiss, W. D. (1998). Brain plasticity in poststroke aphasia: what is the contribution of the right hemisphere?. Brain and language, 64(2), 215-230. 7. Crosson B, Moore AB, Gopinath K, et al. Role of the right and left hemispheres in recovery of function during treatment of intention in aphasia. J Cogn Neurosci. 2005;17:392–406. 8. Johnson, J. P., Meier, E. L., Pan, Y., & Kiran, S. (2019). Treatment-related changes in neural activation vary according to treatment response and extent of spared tissue in patients with chronic aphasia. Cortex, 121, 147-168. 9. Belin, P., Van Eeckhout, P., Zilbovicius, M., Remy, P., FranFois, C., Guillaume, S., ... & Samson, Y. (1991). Recovery from nonfluent aphasia after melodic intonation therapy. Hum Mov Sci, 10, 315-334. 10. Grossi, D., Trojano, L., Chiacchio, L., Soricelli, A., Mansi, L., Postiglione, A., & Salvatore, M. (1991). Mixed transcortical aphasia: Clinical features and neuroanatomical correlates. European neurology, 31(4), 204-211. 11. Rosen, H. J., Petersen, S. E., Linenweber, M. R., Snyder, A. Z., White, D. A., Chapman, L., ... & Corbetta, M. (2000). Neural correlates of recovery from aphasia after damage to left inferior frontal cortex. Neurology, 55(12), 1883-1894. 12. Marcotte, K., & Ansaldo, A. I. (2010, February). The neural correlates of semantic feature analysis in chronic aphasia: discordant patterns according to the etiology. In *Seminars in Speech and Language* (Vol. 31, No. 01, pp. 052-063). © Thieme Medical Publishers. 13. Miura, K., Nakamura, Y., Miura, F., Yamada, I., Takahashi, M., Yoshikawa, A., & Mizobata, T. (1999). Functional magnetic resonance imaging to word generation task in a patient with Broca’s aphasia. Journal of neurology, 246(10), 939-942. 14. ^*^Cherney, L. R., & Small, S. L. (2006). Task-dependent changes in brain activation following therapy for nonfluent aphasia: discussion of two individual cases. *Journal of the International Neuropsychological Society: JINS*, *12*(6), 828. 15. van de Sandt-Koenderman, M. W., Mendez Orellana, C. P., van der Meulen, I., Smits, M., & Ribbers, G. M. (2018). Language lateralisation after melodic intonation therapy: an fMRI study in subacute and chronic aphasia. Aphasiology, 32(7), 765-783. 16. Wierenga CE, Maher LM, Moore AB, et al. Neural substrates of syntactic mapping treatment: an fMRI study of two cases. J Int Neuropsychol Soc. 2006;12:132–146. 17. Thiel, A., Hartmann, A., Rubi-Fessen, I., Anglade, C., Kracht, L., Weiduschat, N., ... & Heiss, W. D. (2013). Effects of noninvasive brain stimulation on language networks and recovery in early poststroke aphasia. Stroke, 44(8), 2240-2246. 18. Benjamin, M. L., Towler, S., Garcia, A., Park, H., Sudhyadhom, A., Harnish, S., ... & Gonzalez Rothi, L. J. (2014). A behavioral manipulation engages right frontal cortex during aphasia therapy. Neurorehabilitation and neural repair, 28(6), 545-553. 19. Griffis, J. C., Nenert, R., Allendorfer, J. B., & Szaflarski, J. P. (2017). Linking left hemispheric tissue preservation to fMRI language task activation in chronic stroke patients. Cortex, 96, 1-18. 20. Kristinsson, S., Yourganov, G., Xiao, F., Bonilha, L., Stark, B. C., Rorden, C., ... & Fridriksson, J. (2019). Brain-derived neurotrophic factor genotype–specific differences in cortical activation in chronic aphasia. Journal of Speech, Language, and Hearing Research, 62(11), 3923-3936. 21. Heiss, W. D., Karbe, H., Weber-Luxenburger, G., Herholz, K., Kessler, J., Pietrzyk, U., & Pawlik, G. (1997). Speech-induced cerebral metabolic activation reflects recovery from aphasia. Journal of the neurological sciences, 145(2), 213-217. 22. Dietz, A., Vannest, J., Maloney, T., Altaye, M., Holland, S., & Szaflarski, J. P. (2018). The feasibility of improving discourse in people with aphasia through AAC: Clinical and functional MRI correlates. Aphasiology, 32(6), 693-719. 23. Hallam, G. P., Thompson, H. E., Hymers, M., Millman, R. E., Rodd, J. M., Ralph, M. A. L., ... & Jefferies, E. (2018). Task-based and resting-state fMRI reveal compensatory network changes following damage to left inferior frontal gyrus. Cortex, 99, 150-165. 24. Wan, C. Y., Zheng, X., Marchina, S., Norton, A., & Schlaug, G. (2014). Intensive therapy induces contralateral white matter changes in chronic stroke patients with Broca’s aphasia. Brain and language, 136, 1-7. 25. Cao, Y., Vikingstad, E. M., George, K. P., Johnson, A. F., & Welch, K. M. A. (1999). Cortical language activation in stroke patients recovering from aphasia with functional MRI. Stroke, 30(11), 2331-2340. 26. Fridriksson, J., Richardson, J. D., Fillmore, P., & Cai, B. (2012). Left hemisphere plasticity and aphasia recovery. Neuroimage, 60(2), 854-863. 27. Meinzer, M., Rodriguez, A. D., & Rothi, L. J. G. (2012). First decade of research on constrained-induced treatment approaches for aphasia rehabilitation. *Archives of Physical Medicine and Rehabilitation*, *93*(1), S35-S45. 28. Thompson, C. K., & den Ouden, D. B. (2008). Neuroimaging and recovery of language in aphasia. *Current Neurology and Neuroscience Reports*, *8*(6), 475. 29. Zhang, H., Chen, Y., Hu, R., Yang, L., Wang, M., Zhang, J., ... & Du, X. (2017). rTMS treatments combined with speech training for a conduction aphasia patient: A case report with MRI study. *Medicine*, *96*(32). 30. Yoon, S. Y., Kim, J. K., An, Y. S., & Kim, Y. W. (2015). Effect of donepezil on wernicke aphasia after bilateral middle cerebral artery infarction: Subtraction analysis of brain F-18 fluorodeoxyglucose positron emission tomographic images. Clinical Neuropharmacology, 38(4), 147-150. 31. Crosson, B., Moore, A. B., McGregor, K. M., Chang, Y. L., Benjamin, M., Gopinath, K., ... & Rothi, L. J. G. (2009). Regional changes in word-production laterality after a naming treatment designed to produce a rightward shift in frontal activity. Brain and language, 111(2), 73-85. | 1. Buckner, R. L., Corbetta, M., Schatz, J., Raichle, M. E., & Petersen, S. E. (1996). Preserved speech abilities and compensation following prefrontal damage. Proceedings of the national Academy of Sciences, 93(3), 1249-1253. 2. Szaflarski, J. P., Griffis, J., Vannest, J., Allendorfer, J. B., Nenert, R., Amara, A. W., ... & Zhou, X. (2018). A feasibility study of combined intermittent theta burst stimulation and modified constraint-induced aphasia therapy in chronic post-stroke aphasia. Restorative Neurology and Neuroscience, 36(4), 503-518. 3. Hund-Georgiadis, M., Lex, U., Norris, D. G., & von Cramon, D. Y. (2000). Cortical reafferentation following left subcortical hemorrhage: a serial functional MR study. Neurology, 55(8), 1227-1231. 4. Crinion, J. T., Warburton, E. A., Lambon-Ralph, M. A., Howard, D., & Wise, R. J. (2006). Listening to narrative speech after aphasic stroke: the role of the left anterior temporal lobe. Cerebral Cortex, 16(8), 1116-1125. 5. Mohr, B., Difrancesco, S., Harrington, K., Evans, S., & Pulvermüller, F. (2014). Changes of right-hemispheric activation after constraint-induced, intensive language action therapy in chronic aphasia: fMRI evidence from auditory semantic processing. Frontiers in human neuroscience, 8. 6. Butler, R. A., Ralph, M. A. L., & Woollams, A. M. (2014). Capturing multidimensionality in stroke aphasia: mapping principal behavioural components to neural structures. Brain, 137(12), 3248-3266. 7. Brownsett, S. L., Warren, J. E., Geranmayeh, F., Woodhead, Z., Leech, R., & Wise, R. J. (2014). Cognitive control and its impact on recovery from aphasic stroke. Brain, 137(1), 242-254. 8. Szaflarski, J. P., Allendorfer, J. B., Banks, C., Vannest, J., & Holland, S. K. (2013). Recovered vs. not-recovered from post-stroke aphasia: the contributions from the dominant and non-dominant hemispheres. Restorative neurology and neuroscience, 31(4), 347. 9. Zahn, R., Drews, E., Specht, K., Kemeny, S., Reith, W., Willmes, K., ... & Huber, W. (2004). Recovery of semantic word processing in global aphasia: a functional MRI study. Cognitive Brain Research, 18(3), 322-336. 10. Mummery, C. J., Ashburner, J., Scott, S. K., & Wise, R. J. (1999). Functional neuroimaging of speech perception in six normal and two aphasic subjects. The Journal of the Acoustical Society of America, 106(1), 449-457. 11. Zahn, R., Huber, W., Drews, E., Specht, K., Kemeny, S., Reith, W., ... & Schwarz, M. (2002). Recovery of semantic word processing in transcortical sensory aphasia: a functional magnetic resonance imaging study. Neurocase,8(5), 376-386. 12. Calvert, G. A., Brammer, M. J., Morris, R. G., Williams, S. C., King, N., & Matthews, P. M. (2000). Using fMRI to study recovery from acquired dysphasia.Brain and language, 71(3), 391-399. 13. Thulborn, K. R., Carpenter, P. A., & Just, M. A. (1999). Plasticity of language-related brain function during recovery from stroke. Stroke, 30(4), 749-754. 14. Davis CH, Harrington G, Baynes K. Intensive semantic intervention in fluent aphasia: a pilot study with fMRI. Aphasiology. 2006;20:59–83. 15. Thompson CK, Fix SC, Gitelman DG, et al. fMRI studies of agrammatic sentence comprehension before and after treatment. Brain Lang. 2000;74:387–391 16. van Oers, C. A., Vink, M., van Zandvoort, M. J., van der Worp, H. B., de Haan, E. H., Kappelle, L. J., ... & Dijkhuizen, R. M. (2010). Contribution of the left and right inferior frontal gyrus in recovery from aphasia. A functional MRI study in stroke patients with preserved hemodynamic responsiveness. Neuroimage, 49(1), 885-893. 17. Leff, A., Crinion, J., Scott, S., Turkheimer, F., Howard, D., & Wise, R. (2002). A physiological change in the homotopic cortex following left posterior temporal lobe infarction. Annals of neurology, 51(5), 553-558. 18. Zahn, R., Schwarz, M., & Huber, W. (2006). Functional activation studies of word processing in the recovery from aphasia. Journal of Physiology-Paris,99(4), 370-385. 19. Wilson, S. M., Yen, M., & Eriksson, D. K. (2018). A n adaptive semantic matching paradigm for reliable and valid language mapping in individuals with aphasia. *Human brain mapping*, *39*(8), 3285-3307. 20. Fernandez, B., Cardebat, D., Demonet, J. F., Joseph, P. A., Mazaux, J. M., Barat, M., & Allard, M. (2004). Functional MRI follow-up study of language processes in healthy subjects and during recovery in a case of aphasia. *Stroke*, *35*(9), 2171-2176. |
| **Coordinates with Speech Baseline** | |
| 1. Pillay, S. B., Gross, W. L., Graves, W. W., Humphries, C., Book, D. S., & Binder, J. R. (2018). The neural basis of successful word reading in aphasia. Journal of Cognitive Neuroscience, 30(4), 514-525. 2. Blasi, V., Young, A. C., Tansy, A. P., Petersen, S. E., Snyder, A. Z., & Corbetta, M. (2002). Word retrieval learning modulates right frontal cortex in patients with left frontal damage. Neuron, 36(1), 159-170. 3. Schönberger, E., Heim, S., Meffert, E., Pieperhoff, P., da Costa Avelar, P., Huber, W., ... & Grande, M. (2014). The neural correlates of agrammatism: Evidence from aphasic and healthy speakers performing an overt picture description task. Frontiers in psychology, 5. 4. Heath, S., McMahon, K. L., Nickels, L. A., Angwin, A., MacDonald, A. D., van Hees, S., ... & Copland, D. A. (2015). An fMRI investigation of the effects of attempted naming on word retrieval in aphasia. Frontiers in Human Neuroscience, 9, 291. 5. Heath, S., McMahon, K. L., Nickels, L., Angwin, A., MacDonald, A. D., van Hees, S., ... & Copland, D. A. (2012). Neural mechanisms underlying the facilitation of naming in aphasia using a semantic task: an fMRI study. BMC neuroscience, 13(1), 98. 6. Fridriksson, J., Hubbard, H. I., Hudspeth, S. G., Holland, A. L., Bonilha, L., Fromm, D., & Rorden, C. (2012). Speech entrainment enables patients with Broca’s aphasia to produce fluent speech. Brain, 135(12), 3815-3829. 7. Nardo, D., Holland, R., Leff, A. P., Price, C. J., & Crinion, J. T. (2017). Less is more: neural mechanisms underlying anomia treatment in chronic aphasic patients. Brain, 140(11), 3039-3054. 8. Nenert, R., Allendorfer, J. B., Martin, A. M., Banks, C., Ball, A., Vannest, J., ... & Szaflarski, J. P. (2017). Neuroimaging correlates of post-stroke aphasia rehabilitation in a pilot randomized trial of constraint-induced aphasia therapy. Medical science monitor: international medical journal of experimental and clinical research, 23, 3489. 9. Schönberger, E., Heim, S., Meffert, E., Pieperhoff, P., da Costa Avelar, P., Huber, W., ... & Grande, M. (2014). The neural correlates of agrammatism: Evidence from aphasic and healthy speakers performing an overt picture description task. Frontiers in psychology, 5, 246. 10. Kurland, J., Pulvermüller, F., Silva, N., Burke, K., & Andrianopoulos, M. (2012). Constrained versus unconstrained intensive language therapy in two individuals with chronic, moderate-to-severe aphasia and apraxia of speech: behavioral and fMRI outcomes. American Journal of Speech-Language Pathology. | 1. Tyler, L. K., Marslen-Wilson, W. D., Randall, B., Wright, P., Devereux, B. J., Zhuang, J., ... & Stamatakis, E. A. (2011). Left inferior frontal cortex and syntax: function, structure and behaviour in patients with left hemisphere damage. Brain, 134(2), 415-431. 2. Gold, B. T., & Kertesz, A. (2000). Right hemisphere semantic processing of visual words in an aphasic patient: an fMRI study. Brain and Language, 73(3), 456-465. 3. Harnish, S. M., Neils-Strunjas, J., Lamy, M., & Eliassen, J. (2008). Use of fMRI in the study of chronic aphasia recovery after therapy: a case study. Topics in Stroke Rehabilitation, 15(5), 468-483. 4. Thompson, C. K., Riley, E. A., den Ouden, D. B., Meltzer-Asscher, A., & Lukic, S. (2013). Training verb argument structure production in agrammatic aphasia: Behavioral and neural recovery patterns. cortex, 49(9), 2358-2376. 5. Specht, K., Zahn, R., Willmes, K., Weis, S., Holtel, C., Krause, B. J., ... & Huber, W. (2009). Joint independent component analysis of structural and functional images reveals complex patterns of functional reorganisation in stroke aphasia. Neuroimage, 47(4), 2057-2063. 6. Tyler, L. K., Wright, P., Randall, B., Marslen-Wilson, W. D., & Stamatakis, E. A. (2010). Reorganization of syntactic processing following left-hemisphere brain damage: does right-hemisphere activity preserve function?. Brain, 133(11), 3396-3408. |
| **Non-Linguistic Functional Task** | |
| N/A | 1. Rochon, E., Leonard, C., Burianova, H., Laird, L., Soros, P., Graham, S., & Grady, C. (2010). Neural changes after phonological treatment for anomia: An fMRI study. Brain and language, 114(3), 164-179. 2. Kurland, J., Naeser, M. A., Baker, E. H., Doron, K., Martin, P. I., Seekins, H. E., ... & Yurgelun-Todd, D. (2004). Test-retest reliability of fMRI during nonverbal semantic decisions in moderate-severe nonfluent aphasia patients. Behavioural neurology, 15(3-4), 87-97. |
| **Coordinates Associated with Acute Aphasia** | |
| 1. Buckner, R. L., Corbetta, M., Schatz, J., Raichle, M. E., & Petersen, S. E. (1996). Preserved speech abilities and compensation following prefrontal damage. Proceedings of the national Academy of Sciences, 93(3), 1249-1253. 2. Qiu, W. H., Wu, H. X., Yang, Q. L., Kang, Z., Chen, Z. C., Li, K., ... & Chen, S. Q. (2017). Evidence of cortical reorganization of language networks after stroke with subacute Broca's aphasia: a blood oxygenation level dependent-functional magnetic resonance imaging study. Neural regeneration research, 12(1), 109. 3. Abutalebi, J., Della Rosa, P. A., Tettamanti, M., Green, D. W., & Cappa, S. F. (2009). Bilingual aphasia and language control: a follow-up fMRI and intrinsic connectivity study. Brain and language, 109(2-3), 141-156. 4. Weiduschat, N., Thiel, A., Rubi-Fessen, I., Hartmann, A., Kessler, J., Merl, P., ... & Heiss, W. D. (2011). Effects of repetitive transcranial magnetic stimulation in aphasic stroke: a randomized controlled pilot study. Stroke, 42(2), 409-415. 5. Geranmayeh, F., Chau, T. W., Wise, R. J., Leech, R., & Hampshire, A. (2017). Domain-general subregions of the medial prefrontal cortex contribute to recovery of language after stroke. Brain, 140(7), 1947-1958. 6. Sreedharan, S., Chandran, A., Yanamala, V. R., Sylaja, P. N., Kesavadas, C., & Sitaram, R. (2019). Self-regulation of language areas using real-time functional MRI in stroke patients with expressive aphasia. Brain Imaging and Behavior, 1-17. | 1. Nenert, R., Allendorfer, J. B., Martin, A. M., Banks, C., Vannest, J., Holland, S. K., ... & Szaflarski, J. P. (2018). Longitudinal fMRI study of language recovery after a left hemispheric ischemic stroke. Restorative neurology and neuroscience, 36(3), 359-385. 2. Altamura, C., Reinhard, M., Vry, M. S., Kaller, C. P., Hamzei, F., Vernieri, F., ... & Saur, D. (2009). The longitudinal changes of BOLD response and cerebral hemodynamics from acute to subacute stroke. A fMRI and TCD study. BMC neuroscience, 10(1), 151. 3. Maiorova, L. A., Martynova, O. V., Fedina, O. N., & Petrushevskii, A. G. (2014). An fMRI Study of Impairments to Speech Perception in Patients with Vascular Sensory Aphasia. Neuroscience and Behavioral Physiology, 44(7), 740-747. |
| **No Whole Brain Analysis (e.g., ROI/VOI coordinates)** | |
| 1. Skipper‐Kallal, L. M., Lacey, E. H., Xing, S., & Turkeltaub, P. E. (2017). Functional activation independently contributes to naming ability and relates to lesion site in post‐stroke aphasia. Human Brain Mapping, 38(4), 2051-2066. 2. Seghier, M. L., Bagdasaryan, J., Jung, D. E., & Price, C. J. (2014). The Importance of Premotor Cortex for Supporting Speech Production after Left Capsular-Putaminal Damage. The Journal of Neuroscience, 34(43), 14338-14348. 3. Connor, L. T., Braby, T. D., Snyder, A. Z., Lewis, C., Blasi, V., & Corbetta, M. (2006). Cerebellar activity switches hemispheres with cerebral recovery in aphasia. Neuropsychologia, 44(2), 171-177. 4. Martin, P. I., Naeser, M. A., Ho, M., Doron, K. W., Kurland, J., Kaplan, J., ... & Pascual-Leone, A. (2009). Overt naming fMRI pre-and post-TMS: Two nonfluent aphasia patients, with and without improved naming post-TMS. Brain and language, 111(1), 20-35. 5. Akinina, Y., Dragoy, O., Ivanova, M. V., Iskra, E. V., Soloukhina, O. A., Petryshevsky, A. G., ... & Dronkers, N. F. (2019). Grey and white matter substrates of action naming. *Neuropsychologia*, *131*, 249-265. | 1. Chu, R., Meltzer, J. A., & Bitan, T. (2018). Interhemispheric interactions during sentence comprehension in patients with aphasia. Cortex, 109, 74-91. (sentence picture matching task) 2. Schofield, T. M., Penny, W. D., Stephan, K. E., Crinion, J. T., Thompson, A. J., Price, C. J., & Leff, A. P. (2012). Changes in auditory feedback connections determine the severity of speech processing deficits after stroke. Journal of Neuroscience, 32(12), 4260-4270. 3. Thompson, C. K., Bonakdarpour, B., & Fix, S. F. (2010). Neural mechanisms of verb argument structure processing in agrammatic aphasic and healthy age-matched listeners. Journal of Cognitive Neuroscience, 22(9), 1993-2011. 4. Sharp, D. J., Turkheimer, F. E., Bose, S. K., Scott, S. K., & Wise, R. J. (2010). Increased frontoparietal integration after stroke and cognitive recovery. Annals of neurology, 68(5), 753-756. 5. Thompson, C. K., den Ouden, D. B., Bonakdarpour, B., Garibaldi, K., & Parrish, T. B. (2010). Neural plasticity and treatment-induced recovery of sentence processing in agrammatism. Neuropsychologia, 48(11), 3211-3227. 6. Sims, J. A., Kapse, K., Glynn, P., Sandberg, C., Tripodis, Y., & Kiran, S. (2016). The relationships between the amount of spared tissue, percent signal change, and accuracy in semantic processing in aphasia. Neuropsychologia, 84, 113-126. 7. Woollams, A. M., Halai, A., & Ralph, M. A. L. (2018). Mapping the intersection of language and reading: the neural bases of the primary systems hypothesis. Brain Structure and Function, 223(8), 3769-3786. |
| **No Functional Task Analysis (e.g., structural, rs-fMRI)** | |
| 1. Basilakos, A., Rorden, C., Bonilha, L., Moser, D., & Fridriksson, J. (2015). Patterns of Poststroke Brain Damage That Predict Speech Production Errors in Apraxia of Speech and Aphasia Dissociate. Stroke, 46(6), 1561-1566. 2. Butler, R. A., Ralph, M. A. L., & Woollams, A. M. (2014). Capturing multidimensionality in stroke aphasia: mapping principal behavioural components to neural structures. Brain, 137(12), 3248-3266. 3. Parkinson, R. B., Raymer, A., Chang, Y. L., FitzGerald, D. B., & Crosson, B. (2009). Lesion characteristics related to treatment improvement in object and action naming for patients with chronic aphasia. Brain and language, 110(2), 61-70. 4. Ripamonti, E., Frustaci, M., Zonca, G., Aggujaro, S., Molteni, F., & Luzzatti, C. (2018). Disentangling phonological and articulatory processing: A neuroanatomical study in aphasia. Neuropsychologia, 121, 175-185. 5. Rosso, C., Perlbarg, V., Valabregue, R., Arbizu, C., Ferrieux, S., Alshawan, B., ... & Samson, Y. (2014). Broca's Area Damage is Necessary but not Sufficient to Induce After-effects of cathodal tDCS on the Unaffected Hemisphere in Post-stroke Aphasia. Brain stimulation, 7(5), 627-635. 6. Geranmayeh, F., Leech, R., & Wise, R. J. (2016). Network dysfunction predicts speech production after left hemisphere stroke. Neurology, 86(14), 1296-1305. 7. Marcotte, K., Perlbarg, V., Marrelec, G., Benali, H., & Ansaldo, A. I. (2013). Default-mode network functional connectivity in aphasia: therapy-induced neuroplasticity. Brain and language, 124(1), 45-55. 8. Yourganov, G., Fridriksson, J., Rorden, C., Gleichgerrcht, E., & Bonilha, L. (2016). Multivariate connectome-based symptom mapping in post-stroke patients: networks supporting language and speech. Journal of Neuroscience, 36(25), 6668-6679. 9. Python, G., Glize, B., & Laganaro, M. (2018). The involvement of left inferior frontal and middle temporal cortices in word production unveiled by greater facilitation effects following brain damage. *Neuropsychologia*, *121*, 122-134. | 1. Robson, H., Grube, M., Ralph, M. A. L., Griffiths, T. D., & Sage, K. (2013). Fundamental deficits of auditory perception in Wernicke's aphasia. Cortex,49(7), 1808-1822. 2. Robson, H., Keidel, J. L., Ralph, M. A. L., & Sage, K. (2012). Revealing and quantifying the impaired phonological analysis underpinning impaired comprehension in Wernicke's aphasia. Neuropsychologia, 50(2), 276-288. 3. Szelag, E., Lewandowska, M., Wolak, T., Seniow, J., Poniatowska, R., Pöppel, E., & Szymaszek, A. (2014). Training in rapid auditory processing ameliorates auditory comprehension in aphasic patients: A randomized controlled pilot study. Journal of the neurological sciences, 338(1), 77-86. 4. DeMarco, A. T., & Turkeltaub, P. E. (2020). Functional anomaly mapping reveals local and distant dysfunction caused by brain lesions. NeuroImage, 116806. 5. Johnson, J. P., Meier, E. L., Pan, Y., & Kiran, S. (2020). Pre-treatment graph measures of a functional semantic network are associated with naming therapy outcomes in chronic aphasia. Brain and Language, 207, 104809. 6. Papoutsi, M., Stamatakis, E. A., Griffiths, J., Marslen-Wilson, W. D., & Tyler, L. K. (2011). Is left fronto-temporal connectivity essential for syntax? Effective connectivity, tractography and performance in left-hemisphere damaged patients. Neuroimage, 58(2), 656-664. 7. Bonilha, L., Hillis, A. E., Hickok, G., Den Ouden, D. B., Rorden, C., & Fridriksson, J. (2017). Temporal lobe networks supporting the comprehension of spoken words. Brain, 140(9), 2370-2380. |
| **Coordinates Related to Treatment** | |
| 1. Fridriksson, J., Bonilha, L., Baker, J. M., Moser, D., & Rorden, C. (2010). Activity in preserved left hemisphere regions predicts anomia severity in aphasia. Cerebral Cortex, 20(5), 1013-1019. 2. Menke, R., Meinzer, M., Kugel, H., Deppe, M., Baumgärtner, A., Schiffbauer, H., ... & Knecht, S. (2009). Imaging short-and long-term training success in chronic aphasia. BMC neuroscience, 10(1), 118. 3. Griffis, J. C., Nenert, R., Allendorfer, J. B., & Szaflarski, J. P. (2016). Interhemispheric plasticity following intermittent theta burst stimulation in chronic poststroke aphasia. Neural Plasticity, 4796906. 4. Darkow, R., Martin, A., Würtz, A., Flöel, A., & Meinzer, M. (2017). Transcranial direct current stimulation effects on neural processing in post‐stroke aphasia. Human brain mapping, 38(3), 1518-1531. 5. Raboyeau G, De Boissezon X, Marie N, et al. Right hemisphere activation in recovery from aphasia: lesion effect or function recruitment? Neurology. 2008;70:290–298. 6. Tabei, K. I., Satoh, M., Nakano, C., Ito, A., Shimoji, Y., Kida, H., ... & Tomimoto, H. (2016). Improved neural processing efficiency in a chronic aphasia patient following melodic intonation therapy: a neuropsychological and functional MRI study. Frontiers in neurology, 7, 148. 7. Purcell, J. J., Wiley, R. W., & Rapp, B. (2019). Re-learning to be different: Increased neural differentiation supports post-stroke language recovery. NeuroImage, 202, 116145. 8. Jungblut, M., Mais, C., Huber, W., Binkofski, F. C., & Schüppen, A. (2020). 5-year course of therapy-induced recovery in chronic non-fluent aphasia-Three single cases. cortex, 132, 147-165. 9. Kiran, S., Meier, E. L., Kapse, K. J., & Glynn, P. (2015). Changes in task-based effective connectivity in language networks following rehabilitation in post-stroke patients with aphasia. Frontiers in Human Neuroscience, 9, 316. 10. Meinzer, M., Flaisch, T., Breitenstein, C., Wienbruch, C., Elbert, T., & Rockstroh, B. (2008). Functional re-recruitment of dysfunctional brain areas predicts language recovery in chronic aphasia. Neuroimage, 39(4), 2038-2046. 11. Marcotte, K., Laird, L., Bitan, T., Meltzer, J. A., Graham, S. J., Leonard, C., & Rochon, E. (2018). Therapy-induced neuroplasticity in chronic aphasia after phonological component analysis: A matter of intensity. Frontiers in neurology, 9, 225. | 1. Higgins, J., Barbieri, E., Wang, X., Mack, J., Caplan, D., Kiran, S., ... & Parrish, T. (2020). Reliability of BOLD signals in chronic stroke‐induced aphasia. European Journal of Neuroscience. 2. Musso, M., Weiller, C., Kiebel, S., Müller, S. P., Bülau, P., & Rijntjes, M. (1999). Training-induced brain plasticity in aphasia. Brain, 122(9), 1781-1790. 3. Chau, A. C., Cheung, R. T. F., Jiang, X., Au-Yeung, P. K., & Li, L. S. (2010). An fMRI study showing the effect of acupuncture in chronic stage stroke patients with aphasia. Journal of acupuncture and meridian studies, 3(1), 53-57. 4. Szaflarski, J. P., Vannest, J., Wu, S. W., DiFrancesco, M. W., Banks, C., & Gilbert, D. L. (2011). Excitatory repetitive transcranial magnetic stimulation induces improvements in chronic post-stroke aphasia. Medical science monitor: international medical journal of experimental and clinical research, 17(3), CR132. 5. Sandberg, C. W., Bohland, J. W., & Kiran, S. (2015). Changes in functional connectivity related to direct training and generalization effects of a word finding treatment in chronic aphasia. Brain and language, 150, 103-116. 6. Barbieri, E., Mack, J., Chiappetta, B., Europa, E., & Thompson, C. K. (2019). Recovery of offline and online sentence processing in aphasia: Language and domain-general network neuroplasticity. Cortex, 120, 394-418. 7. Purcell, J. J., Wiley, R. W., & Rapp, B. (2019). Re-learning to be different: Increased neural differentiation supports post-stroke language recovery. NeuroImage, 202, 116145. 8. Dreyer, F. R., Doppelbauer, L., Büscher, V., Arndt, V., Stahl, B., Lucchese, G., ... & Pulvermüller, F. (2020). Increased Recruitment of Domain-General Neural Networks in Language Processing Following Intensive Language-Action Therapy: fMRI Evidence From People With Chronic Aphasia. American Journal of Speech-Language Pathology, 1-11. |
| **Aphasia Secondary to Non-Left Hemisphere Stroke (e.g., Tumor, TBI, RH Stroke)** | |
| 1. ^*^Tomasino, B., Marin, D., Maieron, M., D'Agostini, S., Medeossi, I., Fabbro, F., ... & Luzzatti, C. (2015). A multimodal mapping study of conduction aphasia with impaired repetition and spared reading aloud. Neuropsychologia, 70, 214-226. 2. Coelho, C., Lê, K., Mozeiko, J., Krueger, F., & Grafman, J. (2012). Discourse production following injury to the dorsolateral prefrontal cortex. Neuropsychologia, 50(14), 3564-3572. |  |
| **Pediatric Stroke (prenatal, perinatal, postnatal up to 18 years)** | |
| 1. Szaflarski, J. P., Allendorfer, J. B., Byars, A. W., Vannest, J., Dietz, A., Hernando, K. A., & Holland, S. K. (2014). Age at stroke determines post-stroke language lateralization. Restorative neurology and neuroscience, 32(6), 733-742. | N/A |
| **Coordinates Compare PWA to Control Group** | |
| 1. Abel, S., Weiller, C., Huber, W., & Willmes, K. (2014). Neural underpinnings for model-oriented therapy of aphasic word production. Neuropsychologia, 57, 154-165. | 1. Baum, S. H., Martin, R. C., Hamilton, A. C., & Beauchamp, M. S. (2012). Multisensory speech perception without the left superior temporal sulcus. Neuroimage, 62(3), 1825-1832. |
| **No PWA Subjects** | |
| 1. Riecker, A., Wildgruber, D., Grodd, W., & Ackermann, H. (2002). Reorganization of speech production at the motor cortex and cerebellum following capsular infarction: a follow-up functional magnetic resonance imaging study. Neurocase, 8(6), 417-423. 2. Baumgaertner, A., Hartwigsen, G., & Roman Siebner, H. (2013). Right‐hemispheric processing of non‐linguistic word features: Implications for mapping language recovery after stroke. Human brain mapping, 34(6), 1293-1305. 3. Rueckert, L., Appollonio, I., Grafman, J., Jezzard, P., Johnson Jr, R., Le Bihan, D., & Turner, R. (1994). Magnetic resonance imaging functional activation of left frontal cortex during covert word production. Journal of neuroimaging: official journal of the American Society of Neuroimaging, 4(2), 67-70. | N/A |
| **Behavioral Data Only** | |
| 1. Goral, M., Naghibolhosseini, M., & Conner, P. S. (2013). Asymmetric inhibitory treatment effects in multilingual aphasia. Cognitive neuropsychology, 30(7-8), 564-577. | 1. Healy, E. W., Moser, D. C., Morrow-Odom, K. L., Hall, D. A., & Fridriksson, J. (2007). Speech perception in MRI scanner noise by persons with aphasia. Journal of Speech, Language, and Hearing Research, 50(2), 323-334. |
| **Analysis of Interest Reported as Part of an Already Included Study** | |
| 1. Baker, J., Rorden, C., & Fridriksson, J. (2010). Using transcranial direct current stimulation (tDCS) to treat stroke patients with aphasia. Stroke; a Journal of Cerebral Circulation, 41(6), 1229–1236. doi:10.1161/STROKEAHA.109.576785 2. Belin, P., Zilbovicius, M., Remy, P., Francois, C., Guillaume, S., Chain, F., ... & Samson, Y. (1996). Recovery from nonfluent aphasia after melodic intonation therapy A PET study. Neurology, 47(6), 1504-1511. | N/A |

Supplementary Table 2. Anatomical locations of peak coordinates and cluster size for each single condition and contrast ALE in PWA from the 33 studies (16 production, 17 comprehension) that also reported control activations for the same tasks.

| **Condition** | **Anatomical Location** | **Peak Coordinates** | **Voxels (mm^3^)** |
| --- | --- | --- | --- |
| Production and Comprehension | Left middle frontal gyrus*, left inferior frontal gyrus (pars orbitalis) | -44, 22, 20 | 12264 |
|  | Left superior frontal gyrus*, right medial frontal gyrus | -6, 8, 52 | 1928 |
|  | Left middle temporal gyrus*, left superior temporal gyrus | -50, -36, -2 | 4824 |
|  | Left superior temporal gyrus* | -50, -8, -10 | 1200 |
|  | Right insula*, right inferior frontal gyrus (pars triangularis, pars orbitalis), right middle frontal gyrus, right precentral gyrus | 32, 20, 4 | 10920 |
|  | Right middle temporal gyrus*, right superior temporal gyrus | 56, -34, 6 | 5040 |
| Production | Left middle frontal gyrus* | -42, 26, 18 | 1232 |
|  | Right precentral gyrus*, right insula | 46, 18, 8 | 4344 |
|  | Right middle temporal gyrus*, right superior temporal gyrus | 56, -34, 6 | 3856 |
| Comprehension | Left inferior frontal gyrus (pars orbitalis*) | -38, 28, -4 | 2544 |
|  | Left middle frontal gyrus* | -46, 20, 20 | 3512 |
|  | Left superior frontal gyrus* | -8, 8, 52 | 904 |
|  | Left precentral gyrus* | -46, -2, 40 | 1616 |
|  | Left middle temporal gyrus*, left superior temporal gyrus | -50, -36, -2 | 3616 |
|  | Left middle temporal gyrus* | -40, -64, 20 | 992 |
|  | Left superior temporal gyrus* | -50, -8, -10 | 2040 |
|  | Right middle frontal gyrus* | 46, 22, 26 | 1232 |
|  | Right claustrum*, right inferior frontal gyrus (pars orbitalis) | 30, 20, 4 | 1944 |
| Conjunction Analysis: Production and Comprehension | Left middle frontal gyrus* | -42, 24, 18 | 784 |
|  | Right inferior frontal gyrus (pars orbitalis*) | 44, 22, 0 | 8 |
|  | Right insula*, right inferior frontal gyrus (pars orbitalis) | 32, 20, 6 | 728 |
| Production > Comprehension | Right superior temporal gyrus* | 56, -30, 12 | 2848 |
| Comprehension > Production | Left inferior frontal gyrus (pars triangularis*, pars orbitalis) | -49, 20.3, 16.5 | 6272 |
|  | Left superior frontal gyrus* | -6, 14, 54 | 848 |
|  | Left precentral gyrus* | -41.8, -2.5, 45.7 | 1616 |
|  | Left middle temporal gyrus* | -54, -39, -4 | 3288 |
|  | Left middle temporal gyrus* | -52.6, -3.7, -14.3 | 2032 |
|  | Left middle temporal gyrus* | -46, -68, 24 | 992 |
|  | Right middle frontal gyrus* | 42, 18, 24 | 776 |
|  | Right putamen (lentiform nucleus)* | 26, 16, 0 | 200 |
| The x, y, z coordinates are in Talairach space and refer to the peak voxel activated in each cluster. All single condition ALEs are thresholded at *p<*0.001 corrected and contrast ALEs at *p=*.05 uncorrected. Asterisks indicate anatomical location of peak voxel. | | | |

Supplementary Table 3. Anatomical locations of peak coordinates and cluster size for each single condition and contrast ALE in controls.

| **Condition** | **Anatomical Location** | **Peak Coordinates** | **Voxels (mm^3^)** |
| --- | --- | --- | --- |
| Production and Comprehension | Left inferior frontal gyrus (pars opercularis*, triangularis, orbitalis), left middle frontal gyrus, left insula | -44, 8, 24 | 13024 |
|  | Left superior frontal gyrus* | -4, 18, 48 | 4288 |
|  | Left precentral gyrus* | -46, 0, 48 | 1096 |
|  | Left middle temporal gyrus* | -54, -42, 4 | 3696 |
|  | Left superior temporal gyrus* | -60, -24, 6 | 1336 |
|  | Right superior temporal gyrus*, right insula, right inferior frontal gyrus (pars triangularis) | 52, 12, -8 | 3680 |
|  | Right superior temporal gyrus*, right middle temporal gyrus | 64, -28, 4 | 2152 |
|  | Left superior parietal lobule*, left inferior parietal lobule | -28, -60, 42 | 1376 |
| Production | Left inferior frontal gyrus (pars opercularis,* pars triangularis),  left middle frontal gyrus, left insula | -46, 8, 24 | 6328 |
|  | Left superior frontal gyrus*, left medial frontal gyrus, left cingulate gyrus, right superior frontal gyrus | -2, 18, 48 | 3464 |
|  | Left middle temporal gyrus*, left superior temporal gyrus | -52, -44, 6 | 1176 |
|  | Left superior temporal gyrus*, left transverse temporal gyrus | -56, -24, 4 | 904 |
|  | Right insula*, right inferior frontal gyrus (pars triangularis) | 38, 22, 0 | 1032 |
|  | Right superior temporal gyrus*, right middle temporal gyrus | 64, -28, 4 | 1800 |
| Comprehension | Left middle frontal gyrus* | -40, 8, 28 | 3104 |
|  | Left inferior frontal gyrus (pars triangularis*, pars orbitalis) | -40, 20, 6 | 2872 |
|  | Left middle temporal gyrus* | -56, -42, 2 | 2760 |
| Conjunction Analysis: Production and Comprehension | Left inferior frontal gyrus (pars orbitalis)* | -42, 8, 26 | 1192 |
|  | Left inferior frontal gyrus (pars triangularis)* | -48, 20, 14 | 296 |
|  | Left inferior frontal gyrus (pars triangularis)* | -38, 22, 8 | 8 |
|  | Left inferior frontal gyrus (pars triangularis)* | -40, 24, 10 | 8 |
|  | Left middle frontal gyrus* | -44, 22, 26 | 224 |
|  | Left middle frontal gyrus* | -42, 20, 22 | 8 |
|  | Left insula* | -36, 20, 6 | 24 |
|  | Left middle temporal gyrus* | -54, -44, 4 | 312 |
| Production > Comprehension | Left middle frontal gyrus*, left inferior frontal gyrus (pars opercularis) | -50, 30, 18 | 744 |
|  | Left medial frontal gyrus*, left superior frontal gyrus | -3.6, -0.8, 60 | 1472 |
|  | Right inferior frontal gyrus (pars triangularis)* | 50, 22, 6 | 336 |
|  | Right superior temporal gyrus*, right middle temporal gyrus | 62, -28, 8 | 704 |
|  | Right superior temporal gyrus* | 48, -38, 10 | 216 |
| Comprehension > Production | Left inferior frontal gyrus (pars orbitalis)* | -42, -26, -10 | 664 |
|  | Left middle temporal gyrus* | -56, -42, -6 | 1048 |
| The x, y, z coordinates are in Talairach space and refer to the peak voxel activated in each cluster. All single condition ALEs are thresholded at *p<*0.001 corrected and contrast ALEs at *p=*.05 uncorrected. Asterisks indicate anatomical location of peak voxel. | | | |

Supplementary Table 4. Anatomical locations of peak coordinates and cluster size for the Broca’s aphasia and Wernicke’s aphasia ALEs for language production tasks.

| **Condition** | **Anatomical Location** | **Peak Coordinates** | **Voxels (mm^3^)** |
| --- | --- | --- | --- |
| Broca’s Aphasia | Left postcentral gyrus* | -42, -16, 48 | 3088 |
|  | Right middle frontal gyrus* | 50, 32, 18 | 752 |
|  | Right medial frontal gyrus* | 4, -8, 68 | 200 |
|  | Right precentral gyrus*, right superior temporal gyrus, right middle temporal gyrus | 54, -4, 30 | 15640 |
| Wernicke’s Aphasia | Left caudate* | -6, 14, 12 | 888 |
|  | Right inferior frontal gyrus (pars opercularis*) | 44, 20, 8 | 1656 |
|  | Right middle temporal gyrus* | 60, -36, 6 | 952 |
| The x, y, z coordinates are in Talairach space and refer to the peak voxel activated in each cluster. All single condition ALEs are thresholded at *p<*.001 corrected. Asterisks indicate anatomical location of peak voxel.  To be included in this analysis, the study needed to explicitly state that the participant had Broca’s or Wernicke’s aphasia. Studies could alternatively provide standardized scores (e.g., *Western Aphasia Battery-Revised*, *Boston Diagnostic Aphasia Examination*) and/or keywords such as “agrammatic” that allowed for subtyping based on a chart or text description. Studies were excluded from this analysis if they included multiple subtypes of aphasia in the analysis or if their descriptions of the aphasia subtype were vague (e.g., non-fluent or expressive aphasia). The included studies are reported in Supplementary Table 6. | | | |

Supplementary Table 5. Anatomical locations of peak coordinates and cluster size for post-hoc analyses dividing the comprehension tasks by sensory modality (auditory versus visual reading) in PWA.

| **Threshold = *p<*.001 corrected** | | | |
| --- | --- | --- | --- |
| Auditory Comprehension | Left inferior frontal gyrus (pars orbitalis)* | -40, 28, -4 | 1552 |
|  | Left middle frontal gyrus* | -46, 22, 20 | 1464 |
|  | Left medial frontal gyrus* | -8, 8, 50 | 1224 |
|  | Left middle temporal gyrus*, left superior temporal gyrus | -50, -38, -2 | 1824 |
|  | Left superior temporal gyrus* | -50, -8, -10 | 2064 |
|  | Right claustrum* | 30, 20, 4 | 1272 |
| Visual Reading Comprehension | N/A |  |  |
| **Threshold = *p<*.001 uncorrected** | | | |
| Auditory Comprehension | Left inferior frontal gyrus (pars orbitalis*) | -40, 28, -4 | 1552 |
|  | Left middle frontal gyrus* | -46, 22, 20 | 1464 |
|  | Left medial frontal gyrus* | -8, 8, 50 | 1224 |
|  | Left superior frontal gyrus* | -4, 30, 56 | 240 |
|  | Left insula* | -46, 6, 16 | 312 |
|  | Left precentral gyrus* | -40, -4, 46 | 272 |
|  | Left middle temporal gyrus*, left superior temporal gyrus | -50, -38, -2 | 1824 |
|  | Left middle temporal gyrus* | -42, -64, 22 | 352 |
|  | Left superior temporal gyrus* | -50, -8, -10 | 2064 |
|  | Left claustrum* | -28, 20, 6 | 312 |
|  | Right middle frontal gyrus* | 46, 24, 26 | 360 |
|  | Right cingulate gyrus* | 6, 8, 42 | 304 |
|  | Right inferior temporal gyrus* | 50, -32, -10 | 328 |
|  | Right middle temporal gyrus* | 54, 10, -28 | 248 |
|  | Right claustrum* | 30, 20, 4 | 1272 |
| Visual Reading Comprehension | Left inferior frontal gyrus (pars orbitalis*) | -32, 30, -8 | 272 |
|  | Left middle frontal gyrus* | -44, 20, 20 | 376 |
|  | Left middle temporal gyrus* | -56, -40, -10 | 520 |
|  | Left middle temporal gyrus* | -38, -66, 20 | 248 |
|  | Left angular gyrus* | -48, -68, 28 | 208 |
|  | Left cuneus* | -22, -78, 20 | 240 |
|  | Right inferior frontal gyrus (pars orbitalis*) | 40, 28, 0 | 240 |
|  | Right middle frontal gyrus* | 48, 18, 22 | 560 |
|  | Right middle occipital gyrus* | 30, -84, 0 | 296 |
| The x, y, z coordinates are in Talairach space and refer to the peak voxel activated in each cluster. Asterisks indicate anatomical location of peak voxel. | | | |

Supplementary Table 6. Studies included in the meta-analysis.

| Study Citation | Task | Subjects in Analysis  (PWA / Controls) | Imaging (Stereotaxic Space) | Number of Foci  (PWA / Controls) |
| --- | --- | --- | --- | --- |
| **Citations with Production Coordinates** | | | | |
| 1. ^j^Postman-Caucheteux, W. A., Birn, R. M., Pursley, R. H., Butman, J. A., Solomon, J. M., Picchioni, D., ... & Braun, A. R. (2010). Single-trial fMRI shows contralesional activity linked to overt naming errors in chronic aphasic patients.*Journal of Cognitive Neuroscience*, *22*(6), 1299-1318. | Overt picture naming > rest | 3^a^ / 4 | fMRI (Talairach) | 36 / 12 |
| 1. ^k^Meinzer, M., Obleser, J., Flaisch, T., Eulitz, C., & Rockstroh, B. (2007). Recovery from aphasia as a function of language therapy in an early bilingual patient demonstrated by fMRI. *Neuropsychologia*, *45*(6), 1247-1256. | Overt picture naming > rest | 1 / 0 | fMRI (Talairach) | 17 / na |
| 1. ^j,k^Meinzer, M., Flaisch, T., Obleser, J., Assadollahi, R., Djundja, D., Barthel, G., & Rockstroh, B. (2006). Brain regions essential for improved lexical access in an aged aphasic patient: a case report. *BMC neurology*, *6*(1), 28. | Overt picture naming > rest | 1 / 0 | fMRI (Talairach) | 17 / na |
| 1. ^c^Léger, A., Demonet, J. F., Ruff, S., Aithamon, B., Touyeras, B., Puel, M., ... & Cardebat, D. (2002). Neural substrates of spoken language rehabilitation in an aphasic patient: an fMRI study. Neuroimage, 17(1), 174-183. | Overt picture naming > rest | 1 / 6 | fMRI (Talairach) | 12 / 5 |
| 1. Abel, S., Weiller, C., Huber, W., Willmes, K., & Specht, K. (2015). Therapy-induced brain reorganization patterns in aphasia. *Brain*, *138*(4), 1097-1112. | Overt picture naming > rest | 14 / 14 | fMRI (MNI SPM) | 20 / na^f^ |
| 1. Lee, Y. S., Zreik, J. T., & Hamilton, R. H. (2017). Patterns of neural activity predict picture-naming performance of a patient with chronic aphasia. Neuropsychologia, 94, 52-60. | Overt picture naming > rest | 1 / 0 | fMRI (MNI SPM) | 9 / na |
| 1. ^j^van Hees, S., McMahon, K., Angwin, A., de Zubicaray, G., & Copland, D. A. (2014). Neural activity associated with semantic versus phonological anomia treatments in aphasia. *Brain and Language*, *129*, 47-57. | Overt picture naming > rest | 7^a^ / 14 | fMRI (MNI SPM) | 76 / 8 |
| 1. ^j,k^Vitali, P., Abutalebi, J., Tettamanti, M., Danna, M., Ansaldo, A. I., Perani, D., ... & Cappa, S. F. (2007). Training-induced brain remapping in chronic aphasia: a pilot study. *Neurorehabilitation and Neural Repair*, *21*(2), 152-160. | Overt picture naming > rest | 1 / 0 | fMRI (MNI SPM) | 12 / na |
| 1. ^j^Skipper-Kallal, L. M., Lacey, E. H., Xing, S., & Turkeltaub, P. E. (2017). Right hemisphere remapping of naming functions depends on lesion size and location in poststroke aphasia. *Neural Plasticity*, *2017*. | Overt + covert picture naming > rest | 39 / 37 | fMRI (MNI FSL) | Overt: 20 / 16  Covert: 14 / 16 |
| 1. ^h^Harvey, D. Y., Podell, J., Turkeltaub, P. E., Faseyitan, O., Coslett, H. B., & Hamilton, R. H. (2017). Functional reorganization of right prefrontal cortex underlies sustained naming improvements in chronic aphasia via repetitive transcranial magnetic stimulation. Cognitive and behavioral neurology: official journal of the Society for Behavioral and Cognitive Neurology, 30(4), 133. | Overt picture naming > meaningless image | 6 / 0 | fMRI (MNI SPM) | 5 / na |
| 1. ^j,k^Marcotte, K., Adrover-Roig, D., Damien, B., de Preaumont, M., Genereux, S., Hubert, M., & Ansaldo, A. I. (2012). Therapy-induced neuroplasticity in chronic aphasia. *Neuropsychologia*, *50*(8), 1776-1786. | Overt picture naming > meaningless image | 7^a^ / 0 | fMRI (MNI SPM) | 49 / na |
| 1. ^j^Fridriksson, J., Morrow-Odom, L., Moser, D., Fridriksson, A., & Baylis, G. (2006). Neural recruitment associated with anomia treatment in aphasia.*Neuroimage*, *32*(3), 1403-1412. | Overt picture naming > meaningless image | 3^a^ / 2 | fMRI (MNI FSL) | 14 / 7 |
| 1. ^k^Fridriksson, J., Moser, D., Bonilha, L., Morrow-Odom, K. L., Shaw, H., Fridriksson, A., ... & Rorden, C. (2007). Neural correlates of phonological and semantic-based anomia treatment in aphasia. *Neuropsychologia*, *45*(8), 1812-1822. | Overt picture naming > meaningless image | 3^a^ / 10 | fMRI (MNI FSL) | 20 / 5 |
| 1. ^j^Fridriksson, J., Baker, J. M., & Moser, D. (2009). Cortical mapping of naming errors in aphasia. *Human Brain Mapping*, *30*(8), 2487-2498. | Overt picture naming > meaningless image | 11 / 10^b^ | fMRI (MNI FSL) | 8 / na |
| 1. ^c,h,j^Sebastian, R., & Kiran, S. (2011). Task-modulated neural activation patterns in chronic stroke patients with aphasia. Aphasiology, 25(8), 927-951. | Overt picture naming > meaningless image | 8^a^ / 8 | fMRI (MNI FSL) | 62 / 11 |
| 1. ^h^de Boissezon, X., Démonet, J. F., Puel, M., Marie, N., Raboyeau, G., Albucher, J. F., ... & Cardebat, D. (2005). Subcortical aphasia: a longitudinal PET study. Stroke, 36(7), 1467-1473. | Overt noun + verb generation > rest | 6 / 0 | PET (MNI SPM) | 11 / na |
| 1. ^h^De, X. B., Marie, N., Castel-Lacanal, E., Marque, P., Bezy, C., Gros, H., ... & Demonet, J. F. (2009). Good recovery from aphasia is also supported by right basal ganglia: a longitudinal controlled PET study. EJPRM-ESPRM 2008 award winner. European Journal of Physical and Rehabilitation Medicine, 45(4), 547-558. | Overt noun + verb generation > rest | 13 / 0 | PET (MNI SPM) | 13 / na |
| 1. Cardebat, D., Démonet, J. F., de Boissezon, X., Marie, N., Marié, R. M., Lambert, J., ... & Puel, M. (2003). Behavioral and Neurofunctional Changes Over Time in Healthy and Aphasic Subjects A PET Language Activation Study. *Stroke*, *34*(12), 2900-2906. | Overt noun + verb generation > rest | 8 / 6 | PET (MNI SPM) | 11 / 13 |
| 1. Warburton, E., Price, C. J., Swinburn, K., & Wise, R. J. (1999). Mechanisms of recovery from aphasia: evidence from positron emission tomography studies.*Journal of Neurology, Neurosurgery & Psychiatry*, *66*(2), 155-161. | Covert verb generation > rest | 5^a^ / 9 | PET (Talairach) | 27 / 14 |
| 1. ^k^Perani, D., Cappa, S. F., Tettamanti, M., Rosa, M., Scifo, P., Miozzo, A., ... & Fazio, F. (2003). A fMRI study of word retrieval in aphasia. *Brain and Language*,*85*(3), 357-368. | Covert word generation > rest | 4^a^ / 6 | fMRI (MNI SPM) | 52 / 38 |
| 1. Allendorfer, J. B., Kissela, B. M., Holland, S. K., & Szaflarski, J. P. (2012). Different patterns of language activation in post-stroke aphasia are detected by overt and covert versions of the verb generation fMRI task. *Medical Science Monitor: International Medical Journal ff Experimental and Clinical Research*,*18*(3), CR135. | Covert verb generation > finger tapping | 16 / 32 | fMRI (Talairach) | 7 / 8 |
| 1. ^c^Eaton, K. P., Szaflarski, J. P., Altaye, M., Ball, A. L., Kissela, B. M., Banks, C., & Holland, S. K. (2008). Reliability of fMRI for studies of language in post-stroke aphasia subjects. Neuroimage, 41(2), 311-322. | Covert verb generation > finger tapping | 4 / 4 | fMRI (Talairach) | 5 / 8 |
| 1. ^k^Weiller, C., Isensee, C., Rijntjes, M., Huber, W., Müller, S., Bier, D., ... & Diener, H. C. (1995). Recovery from Wernicke's aphasia: a positron emission tomographic study. Annals of Neurology, 37(6), 723-732. | Covert verb generation > rest &  Covert repetition > rest | 6^e^ / 6 | PET (Talairach) | Verb Gen: 7 / 7  Repetition: 5 / 4 |
| 1. Berthier, M. L., Walsh, S. F., Dávila, G., & Nabrozidis, A. (2013). Dissociated repetition deficits in aphasia can reflect flexible interactions between left dorsal and ventral streams and gender-dimorphic architecture of the right dorsal stream. *Frontiers in Human Neuroscience*, *7*. | Covert word repetition > rest | 2^a^ / 0 | fMRI (MNI SPM) | 31 / na |
| 1. Kakuda, W., Abo, M., Kaito, N., Watanabe, M., & Senoo, A. (2010). Functional MRI-based therapeutic rTMS strategy for aphasic stroke patients: a case series pilot study. *International Journal of Neuroscience*, *120*(1), 60-66. | Overt word repetition > rest | 4^ae^ / 0 | fMRI (Talairach) | 25 / na |
| 1. ^k^Abo, M., Senoo, A., Watanabe, S., Miyano, S., Doseki, K., Sasaki, N., ... & Yonemoto, K. (2004). Language-related brain function during word repetition in post-stroke aphasics. *Neuroreport*, *15*(12), 1891-1894. | Overt word repetition > rest | 2^a^ / 6 | fMRI (Talairach) | 15 / 20 |
| 1. ^c, k^Haldin, C., Acher, A., Kauffmann, L., Hueber, T., Cousin, E., Badin, P., ... & Jaillard, A. (2018). Speech recovery and language plasticity can be facilitated by Sensori-Motor Fusion training in chronic non-fluent aphasia. A case report study. Clinical Linguistics & Phonetics, 32(7), 595-621. | Overt syllable repetition > white noise | 1 / 0 | fMRI (MNI SPM) | 16 / na |
| 1. ^c^Richter, M., Miltner, W. H., & Straube, T. (2008). Association between therapy outcome and right-hemispheric activation in chronic aphasia. Brain, 131(5), 1391-1401. | Covert word stem completion > rest | 16 / 8 | fMRI (Talairach) | 5 / 7 |
| 1. Blank, S. C., Bird, H., Turkheimer, F., & Wise, R. J. (2003). Speech production after stroke: the role of the right pars opercularis. *Annals of neurology*, *54*(3), 310-320. | Overt spontaneous language production > rest | 14 / 12 | PET (MNI SPM) | 19 / 11 |
| **Citations with Comprehension Coordinates** | | | | |
| 1. ^h,i^Wright, P., Stamatakis, E. A., & Tyler, L. K. (2012). Differentiating hemispheric contributions to syntax and semantics in patients with left-hemisphere lesions. Journal of Neuroscience, 32(24), 8149-8157. | Auditory sentence listening (word monitoring task) > rest | 21 / 21 | fMRI (MNI SPM) | 8 / 3 |
| 1. Mattioli, F., Ambrosi, C., Mascaro, L., Scarpazza, C., Pasquali, P., Frugoni, M., ... & Gasparotti, R. (2014). Early Aphasia Rehabilitation Is Associated With Functional Reactivation of the Left Inferior Frontal Gyrus A Pilot Study. Stroke, 45(2), 545-552. | Auditory sentence listening (determine whether sentence was semantically correct or incorrect) > rest | 6 / 10 | fMRI (Talairach) | 6 / 7 |
| 1. Stockert, A., Wawrzyniak, M., Klingbeil, J., Wrede, K., Kümmerer, D., Hartwigsen, G., ... & Saur, D. (2020). Dynamics of language reorganization after left temporo-parietal and frontal stroke. Brain. | Auditory sentence listening (determine whether sentence was semantically correct or incorrect) > spectrally rotated sentences | 34 / 17 | fMRI (MNI SPM) | 19 / 17 |
| 1. Saur, D., Lange, R., Baumgaertner, A., Schraknepper, V., Willmes, K., Rijntjes, M., & Weiller, C. (2006). Dynamics of language reorganization after stroke. Brain, 129(6), 1371-1384. | Auditory sentence listening (determine whether sentence was semantically correct or incorrect) > spectrally rotated sentences | 14 / 14 | fMRI (MNI SPM) | 7 / 10 |
| 1. Crinion, J., & Price, C. J. (2005). Right anterior superior temporal activation predicts auditory sentence comprehension following aphasic stroke. Brain, 128(12), 2858-2871. | Auditory sentence listening (passive listening) > spectrally rotated sentences | 17^d^ / 18 | fMRI (Talairach) | 16 / 19 |
| 1. Sahuquillo, J., Radoi, A., Benejam, B., Junqué, C., Fernández-Espejo, D., & Poca, M. A. (2013). Brain activation during speech perception in a patient with a massive left hemisphere infarction. Brain injury, 27(12), 1470-1474. | Auditory sentence listening (passive listening) > spectrally rotated sentences | 1 / 0 | fMRI (MNI SPM) | 5 / na |
| 1. Warren, J. E., Crinion, J. T., Ralph, M. A. L., & Wise, R. J. (2009). Anterior temporal lobe connectivity correlates with functional outcome after aphasic stroke. Brain, awp270. | Auditory sentence listening (passive listening) > spectrally rotated sentences | 16 / 11 | PET (MNI SPM) | 12 / 11 |
| 1. ^c^Eaton, K. P., Szaflarski, J. P., Altaye, M., Ball, A. L., Kissela, B. M., Banks, C., & Holland, S. K. (2008). Reliability of fMRI for studies of language in post-stroke aphasia subjects. Neuroimage, 41(2), 311-322. | Auditory semantic decision > tone decision | 4 / 4 | fMRI (Talairach) | 9 / 12 |
| 1. ^h^Griffis, J. C., Nenert, R., Allendorfer, J. B., Vannest, J., Holland, S., Dietz, A., & Szaflarski, J. P. (2017). The canonical semantic network supports residual language function in chronic post‐stroke aphasia. Human brain mapping, 38(3), 1636-1658. | Auditory semantic decision > tone decision | 43 / 43 | fMRI (MNI SPM) | 13 / 6 |
| 1. ^h^Hartwigsen, G., Stockert, A., Charpentier, L., Wawrzyniak, M., Klingbeil, J., Wrede, K., ... & Saur, D. (2020). Short-term modulation of the lesioned language network. Elife, 9, e54277. | Auditory semantic & phonological decision > rest | 12 / 0 | fMRI (MNI SPM) | 20 / na |
| 1. ^h^Sharp, D. J., Scott, S. K., & Wise, R. J. (2004). Retrieving meaning after temporal lobe infarction: the role of the basal language area. Annals of Neurology: Official Journal of the American Neurological Association and the Child Neurology Society, 56(6), 836-846. | PWA: auditory semantic decision > rest | 9 / 0^g^ | PET (MNI SPM) | 3 / na |
| 1. ^c,h^Sebastian, R., & Kiran, S. (2011). Task-modulated neural activation patterns in chronic stroke patients with aphasia. Aphasiology, 25(8), 927-951. | Visual semantic decision > object size decision | 8^a^ / 8 | fMRI (MNI FSL) | 27 / 4 |
| 1. Sandberg, C., & Kiran, S. (2014). Analysis of abstract and concrete word processing in persons with aphasia and age-matched neurologically healthy adults using fMRI. Neurocase, 20(4), 361-388. | Visual semantic decision > rest | 3^a^ / 3 | fMRI (MNI SPM) | 441 / 70 |
| 1. ^k^Robson, H., Zahn, R., Keidel, J. L., Binney, R. J., Sage, K., & Ralph, M. A. L. (2014). The anterior temporal lobes support residual comprehension in Wernicke’s aphasia. Brain, 137(3), 931-943. | Visual semantic decision > location of scrambled pictures | 12 / 12 | fMRI (MNI SPM) | 11 / 7 |
| 1. ^c^Richter, M., Miltner, W. H., & Straube, T. (2008). Association between therapy outcome and right-hemispheric activation in chronic aphasia. Brain, 131(5), 1391-1401. | Visual single word reading > rest | 16 / 8 | fMRI (Talairach) | 7 / 4 |
| 1. Tuomiranta, L. M., Camara, E., Walsh, S. F., Ripolles, P., Saunavaara, J. P., Parkkola, R., ... & Laine, M. (2014). Hidden word learning capacity through orthography in aphasia. cortex, 50, 174-191. | Visual word & pseudoword reading > meaningless characters | 1 / 7 | fMRI (MNI SPM) | 8 / 5 |
| 1. Price, C. J., Warburton, E. A., Moore, C. J., Frackowiak, R. S. J., & Friston, K. J. (2001). Dynamic diaschisis: anatomically remote and context-sensitive human brain lesions. Journal of Cognitive Neuroscience, 13(4), 419-429. | Study 1: Visual word reading > meaningless consonant strings | Study 1: 4 / 15 | PET (Talairach) | Study 1: 1 / 4 |
| 1. Price, C. J., Mummery, C. J., Moore, C. J., Frackowiak, R. S. J., & Friston, K. J. (1999). Delineating necessary and sufficient neural systems with functional imaging studies of neuropsychological patients. Journal of Cognitive Neuroscience, 11(4), 371-382. | Visual word semantic decision > visual size decision | 1 / 6 | PET (Talairach) | 10 / 16 |
| 1. Szaflarski, J. P., Eaton, K., Ball, A. L., Banks, C., Vannest, J., Allendorfer, J. B., ... & Holland, S. K. (2011). Poststroke aphasia recovery assessed with functional magnetic resonance imaging and a picture identification task. Journal of Stroke and Cerebrovascular Diseases, 20(4), 336-345. | Visual word picture matching > same/different picture judgment | 4 / 4 | fMRI (Talairach) | 3 / 6 |
| 1. ^c^Léger, A., Demonet, J. F., Ruff, S., Aithamon, B., Touyeras, B., Puel, M., ... & Cardebat, D. (2002). Neural substrates of spoken language rehabilitation in an aphasic patient: an fMRI study. Neuroimage, 17(1), 174-183. | Visual word-picture rhyming task > rest | 1 / 6 | fMRI (Talairach) | 12 / 4 |
| 1. ^c,k^Haldin, C., Acher, A., Kauffmann, L., Hueber, T., Cousin, E., Badin, P., ... & Jaillard, A. (2018). Speech recovery and language plasticity can be facilitated by Sensori-Motor Fusion training in chronic non-fluent aphasia. A case report study. Clinical Linguistics & Phonetics, 32(7), 595-621. | Visual word-word rhyming task > rest | 1 / 0 | fMRI (MNI SPM) | 15 / na |
| ^a^ Participants are entered into the analyses as individuals.  ^b^ Control data is reported as part of another study already included in the analysis (Fridriksson et al., 2007).  ^c^ Study contains both production and comprehension tasks/coordinates.  ^d^ One PWA was four-months post-stroke at time of scan.  ^e^ One PWA was five-months post-stroke at time of scan.  ^f^ Coordinates are control > PWA  ^g^ Control task did not meet inclusion criteria; speech > speech baseline  ^h^ Identified by cross-referencing Wilson & Schneck (2021)  ^i^ Three participants had aphasia secondary to surgery.  ^j^ Extracted coordinates associated with picture naming behavioral performance.  ^k^ Study included in the aphasia diagnosis sub-analysis. | | | | |

Supplementary Figure 1. Representative sagittal slices for the language production ALEs in Broca’s aphasia and Wernicke’s aphasia (*p<.*001 corrected).

Supplementary Figure 2. Representative sagittal slices for the production > comprehension and comprehension > production ALEs in PWA from the 33 studies that also reported control activations for the same task (*p=.*05 uncorrected). Only PWA data is shown.

Supplementary Figure 3. Representative sagittal slices for the **(A)** picture naming and word generation, and **(B**) semantic decision and auditory sentence listening ALEs (*p<*.001 corrected). Only PWA data is depicted. ALEs were performed on the subset of tasks that included both PWA and control data. Sample size denotes the number of tasks included in the ALE.
