# Supplementary figures and images for: Neural Resources Supporting Language Production vs. Comprehension in Chronic Post-stroke Aphasia: A Meta-Analysis Using Activation Likelihood Estimates
Source: Front Hum Neurosci. 2021 Oct 25;15:680933. doi: 10.3389/fnhum.2021.680933 (PMC8572938; doi:10.3389/fnhum.2021.680933)

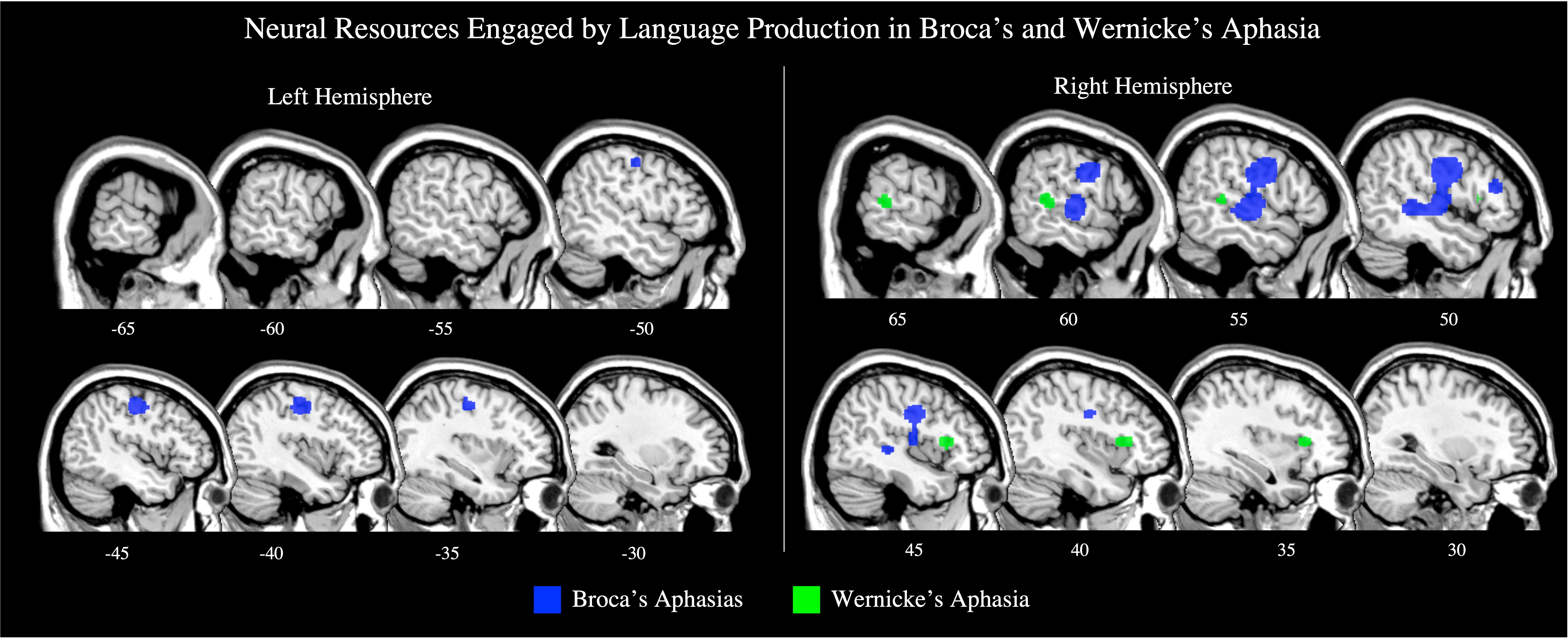

Supplement: Supplementary file 2 [file Image_1.jpg]

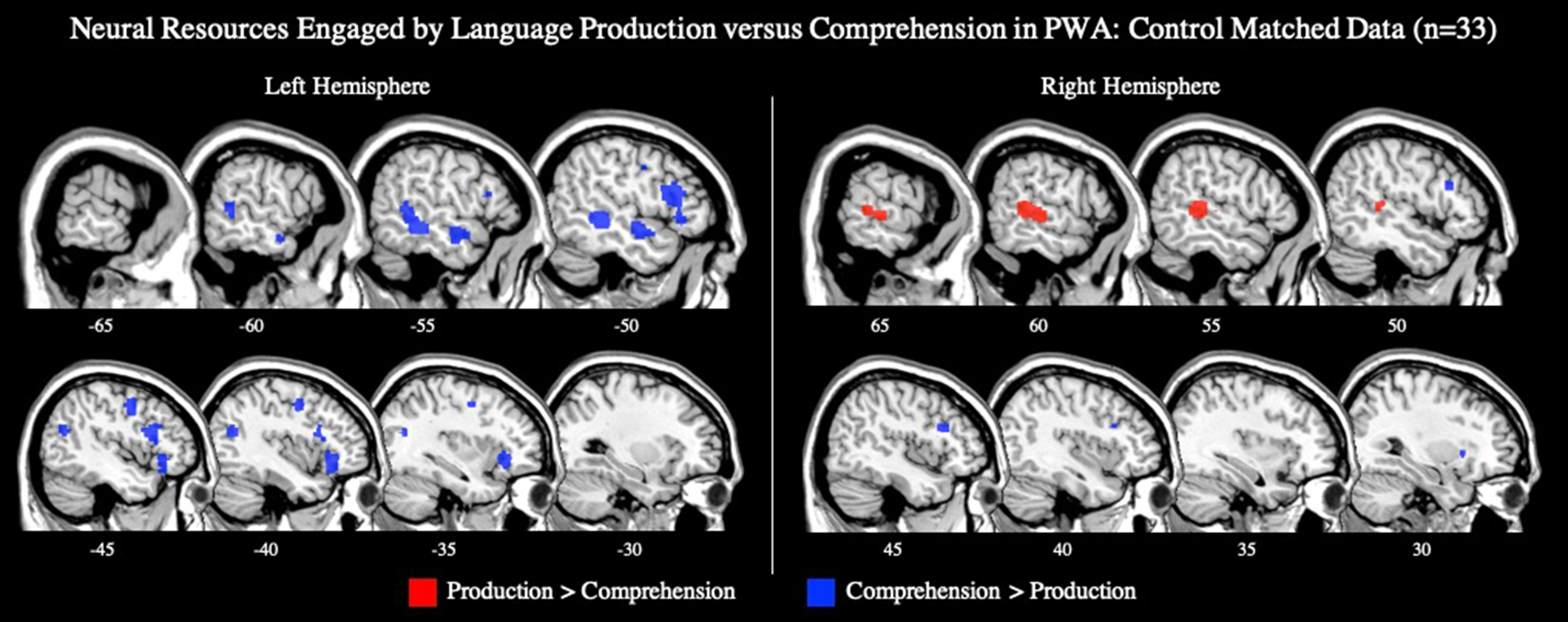

Supplement: Supplementary file 3 [file Image_2.JPEG]

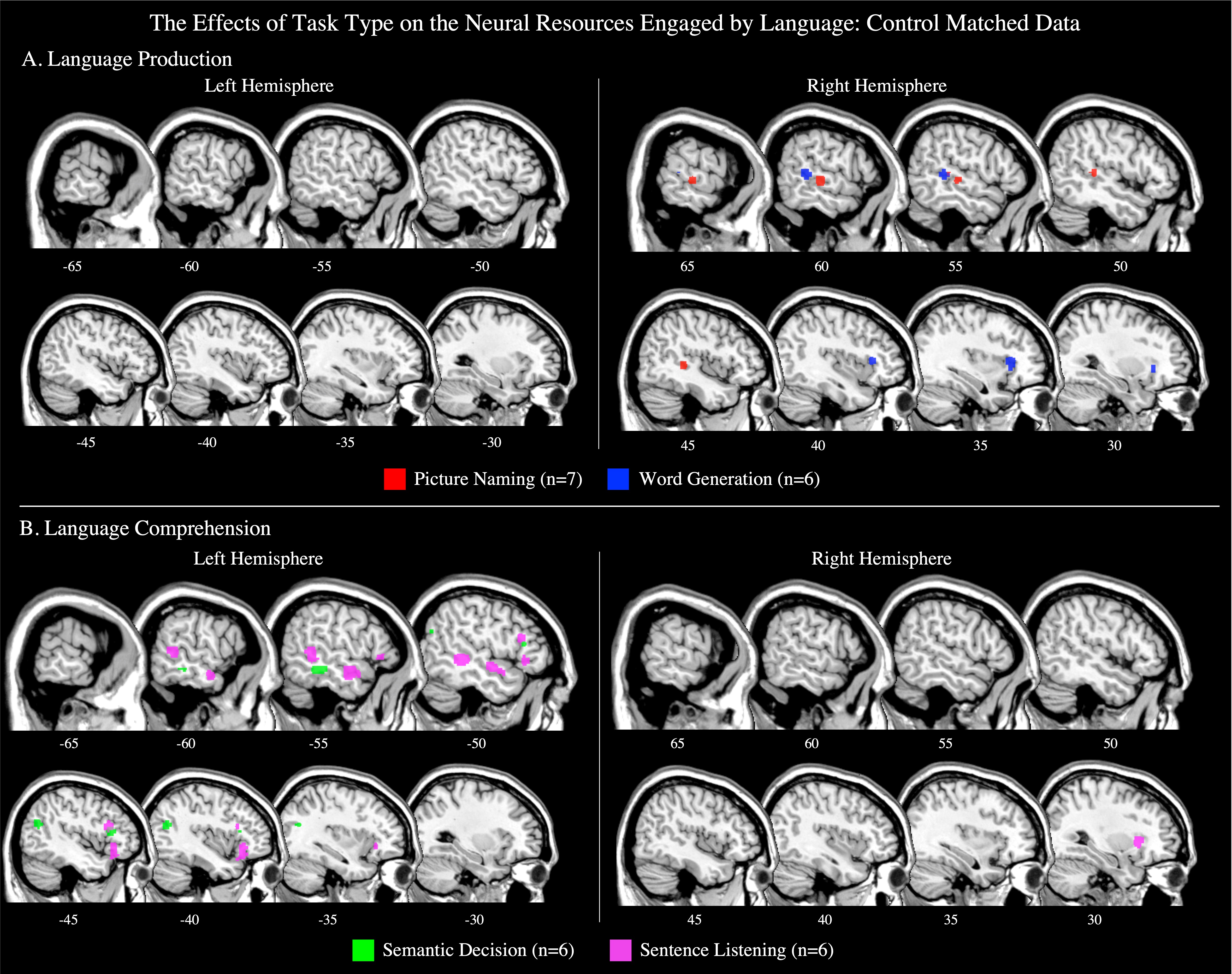

Supplement: Supplementary file 4 [file Image_3.JPEG]
